# Comparing Early Transcriptomic Responses of 18 Soybean (*Glycine max*) Genotypes to Iron Stress

**DOI:** 10.3390/ijms222111643

**Published:** 2021-10-28

**Authors:** Daniel R. Kohlhase, Chantal E. McCabe, Asheesh K. Singh, Jamie A. O’Rourke, Michelle A. Graham

**Affiliations:** 1 Department of Agronomy, Iowa State University, Ames, IA 50011, USA; kohlhase@iastate.edu (D.R.K.); singhak@iastate.edu (A.K.S.); 2 U.S. Department of Agriculture (USDA)—Agricultural Research Service (ARS), Corn Insects and Crop Genetics Research Unit, Ames, IA 50011, USA; McCabe.Chantal@mayo.edu

**Keywords:** *Glycine max*, soybean, iron deficiency chlorosis, abiotic stress, RNA-seq, comparative transcriptomics

## Abstract

Iron deficiency chlorosis (IDC) is an abiotic stress that negatively affects soybean (*Glycine max* [L.] Merr.) production. Much of our knowledge of IDC stress responses is derived from model plant species. Gene expression, quantitative trait loci (QTL) mapping, and genome-wide association studies (GWAS) performed in soybean suggest that stress response differences exist between model and crop species. Our current understanding of the molecular response to IDC in soybeans is largely derived from gene expression studies using near-isogenic lines differing in iron efficiency. To improve iron efficiency in soybeans and other crops, we need to expand gene expression studies to include the diversity present in germplasm collections. Therefore, we collected 216 purified RNA samples (18 genotypes, two tissue types [leaves and roots], two iron treatments [sufficient and deficient], three replicates) and used RNA sequencing to examine the expression differences of 18 diverse soybean genotypes in response to iron deficiency. We found a rapid response to iron deficiency across genotypes, most responding within 60 min of stress. There was little evidence of an overlap of specific differentially expressed genes, and comparisons of gene ontology terms and transcription factor families suggest the utilization of different pathways in the stress response. These initial findings suggest an untapped genetic potential within the soybean germplasm collection that could be used for the continued improvement of iron efficiency in soybean.

## 1. Introduction

Iron deficiency chlorosis (IDC) in soybean (*Glycine max* [L.] Merr.) is characterized by interveinal chlorosis, stunted growth, and yield loss. IDC is typically found in soybeans grown throughout the North Central U.S., where a high pH (>7.2) and calcareous soils limit iron availability, resulting in IDC development [1]. Soil properties and genetic differences between lines create a variability in iron stress tolerance [2,3,4,5]. Froehlich and Fehr (1981) demonstrated the genotypic variability of the IDC response among 15 soybean varieties, finding that each one point change on the IDC visual rating scale (1–5) correlated to an approximately 20% yield loss at the end of the season [3]. Using the 2020 median price of soybean, the estimated economic loss due to IDC in the North Central U.S. would be approximately 117 million USD [1]. Due to the high potential for yield loss associated with IDC, we must improve our understanding of iron stress responses in order to keep economic losses to a minimum.

A collective effort to improve our ability to breed for iron efficiency has resulted in a strong research foundation addressing the genetics of iron utilization and crop stress adaptations. Weiss (1943) was the first to suggest a single dominant gene underlying the efficiency of iron utilization in soybean [6]. Cianzio and Fehr (1980) justified the variation in iron stress responses by suggesting that modifying genes accompany major quantitative trait loci (QTL) [7]. Since then, multiple genetic studies have provided more evidence supporting the idea of multiple genes controlling iron efficiency [8,9,10,11,12]. Diers et al. [13] first mapped an iron efficiency QTL using an early soybean genetic map. Later, Lin et al. [9] mapped an iron efficiency QTL using two different mapping populations: in one population, a number of minor effect QTL were associated with iron efficiency, whereas, in the other population, 68–73% of variance associated with iron efficiency was mapped to a single QTL. Following the publication of the soybean genome, Severin et al. [14] narrowed the location of this major QTL on soybean chromosome Gm03 using an introgression mapping of near-isogenic lines (NILs) Clark (iron stress tolerant) and IsoClark (iron stress susceptible), and the iron inefficiency donor T203 (iron stress susceptible). Peiffer et al. [15] used introgression and QTL mapping to narrow the QTL within the introgressed region even further. Recently, Assefa et al. [12] performed a genome-wide association study, characterizing IDC tolerance in 460+ soybean lines using multiple phenotyping methods and timepoints to evaluate IDC symptoms in the field and in hydroponics. This analysis split this historical QTL into four discrete linkage blocks, each containing candidate iron stress responsive genes. It is still unknown if different combinations of theses linkage blocks can be associated with differences in the stress response and gene expression patterns of soybeans responding to iron stress.

Continued improvements of sequencing technologies allow for scientists to examine genome-wide expression differences in response to stress within any soybean genotypes of interest. Initial soybean gene expression studies using Clark and IsoClark identified responses after 14 days of iron stress, including genes involved in general stress responses, iron uptake/homeostasis, and DNA repair/replication [16,17,18]. Moran Lauter et al. [19] utilized RNA-seq to study the early transcriptional response (1 h and 6 h after iron stress) in the leaf and root tissue of Clark. Similar to the previous gene expression studies, Moran Lauter et al. found genes involved in what are now considered the hallmarks of the Clark iron stress response: a defense response, iron homeostasis, and DNA replication/methylation. Recently, Moran Lauter et al. [20] found Clark responds to iron stress as early as 30 min after stress. In addition, a shift in gene expression from root to shoot was observed 30–120 min after stress onset. This shift was attributed to the movement of a novel stress signal. Atencio et al. [21] examined Clark and IsoClark responses two and ten days after iron stress, observing an ebb and flow in the gene expression across these same pathways. Remarkably, significant differences in the leaf chlorophyll content could be detected at two days of iron stress.

Thus far, the majority of iron stress studies in both model and crop species have been restricted to a few genotypes of interest. Soybean, however, has evolved around the world in different environments and soil conditions. In order to identify and characterize the full breadth of the soybean iron stress response, including novel iron stress tolerance mechanisms, we need to characterize iron stress responses in multiple genotypes. In this study, the objective was to compare early differential gene expression patterns of soybeans with varying iron efficiencies. We selected 18 lines from the Assefa et al. [12] GWAS panel for iron stress response gene expression analyses. We used RNA sequencing on leaf and root tissue collected 60 min after growing plants in either iron-deficient or iron-sufficient hydroponic solutions. After testing for differential expression, we found a rapid and varied response to iron stress across genotypes. While genome-wide association studies and transcriptomic studies have separately proven to be very useful tools in identifying stress or developmental genes and regions of interest, we must leverage both research tools simultaneously to increase our understanding of IDC responses in soybean. Leveraging diversity found in the soybean germplasm collection can be used to enhance breeding efforts and develop a greater tolerance to nutrient stresses, such as IDC.

## 2. Results

### 2.1. Clustering Genotypes into Efficient and Inefficient Classes Based on Phenotypic Data

**Figure 1:**
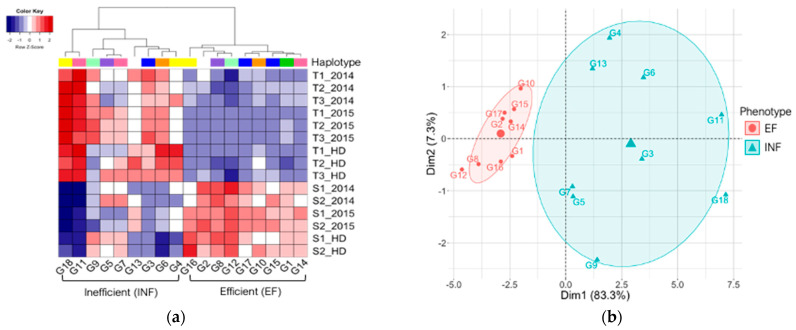
Clustering of 18 soybean genotypes based on phenotypic response to iron stress. (**a**) Iron deficiency chlorosis (IDC) ratings and soil plant analysis development (SPAD) readings from Assefa et al. (2020) were used to generate a heatmap using row z-scores (standard deviation from the mean within a row). IDC ratings were collected at three growth stages (T1, T2, T3) and SPAD readings were collected at two growth stages (S1, S2) across two field seasons (2014, 2015) and in greenhouse hydroponics (HD). Genotypes ordered based on hierarchical clustering revealed two major groups of soybean genotypes, iron–efficient (EF) and iron–inefficient (INF). Haplotypes were identified from sequences within the historic iron deficiency chlorosis (IDC) quantitative trait loci (QTL) on chromosome Gm03. Matching haplotypes are indicated in the color bar above the heatmap. (**b**) Principal component analysis (PCA) was performed on the same data used to generate the heatmap. The two major groups of soybean genotypes revealed by hierarchical clustering, EF and INF, were also seen in the PCA and are shown in red and blue, respectively.

Phenotypic data provided by Assefa et al. [12] were first clustered using all years, growth stages, and environments (Figure 1, Supplementary File S1). The dendrogram from hierarchical clustering shows a distinct break between two clusters, each containing nine genotypes (Figure 1a). To visualize SPAD readings and IDC ratings in the same heatmap, we standardized the phenotypic values in a given growth stage and environment by using z-scores, converting raw values to standard deviations from the mean. Using z-scores, opposite phenotypic ratings for each trait were easily distinguished into two clusters. The cluster with genotypes G1, G2, G8, G10, G12, G14, G15, G16, and G17 (Clark) generally received low IDC ratings and high SPAD readings and will be denoted as the iron-efficient (EF) group. The cluster with genotypes G3, G4, G5, G6, G7, G9, G11, G13, and G18 (IsoClark) generally received high IDC ratings and low SPAD readings and will be denoted as the iron-inefficient (INF) group. Within each cluster, two subgroups could be identified. In the EF group, G2, G8, G12, and G16 were the best performing lines, whereas, in the INF group, G11 and G18 were the worst performing lines. Interestingly, a greater variation of phenotypic scores between years and environments was seen among the INF group, specifically, G5, G7, and G9. The only two genotypes that consistently showed similar phenotypic scores were G11 and G18 of the INF group. Principal component analysis (PCA) was also used to cluster the genotypes (Figure 1b). The first two principal components explained 90.6% of the variance, (83.3% and 7.3%, respectively). In the PCA plot, the genotypes clustered into the same two groups defined using the hierarchical clustering. Again, we saw that the INF group contained more variation than the EF group based on the distribution of the genotypes on the PCA plot.

When selecting the genotypes used in this study, we intentionally selected genotypes with eight haplotype combinations for the three IDC QTL linkage blocks identified by Assefa et al. [12] that corresponded to the narrowed introgressed region identified by Peiffer et al. [15]. For seven of the eight combinations, we selected the two genotypes with the highest and lowest iron stress tolerance, based on three timepoints for visual ratings and two timepoints for SPAD readings [12]. For the eighth combination, only seeds from a single EF genotype was available. Clark (G17, EF) and IsoClark (G18, INF) were then added to the correct haplotype combinations as internal controls. Finally, for one haplotype group, a third genotype was added, representing the average IDC ratings for the group. We found that genotypes clustered by IDC phenotype, not by haplotype, suggesting other genomic regions must impact the IDC tolerance.

### 2.2. Identification of Differentially Expressed Genes in Early Response to IDC Stress

From the 216 purified RNA samples (eighteen genotypes, two tissue types, two iron treatments, three replicates) that were sent to the Iowa State DNA sequencing facility, approximately 6.2 billion raw reads were produced. The sequences were filtered and mapped to the soybean reference genome, as outlined in the materials and methods. The number of mapped reads varied from 5644 to 186,296,039, with nine samples (eight in leaves and one in roots) containing fewer than five million mapped reads (Supplementary File S2). Using FastQ Screen [22] to examine the quality of the reads, along with the unusually low numbers of mapped reads for some samples, raised concerns about the global coverage and depth of sequencing for nine samples. Two genotypes (G3, G15) each had two replicates with fewer than five million mapped reads in leaf tissue samples under sufficient iron conditions. Similarly, genotype (G9) had three replicates with fewer than five million mapped reads in leaf tissue samples under sufficient iron conditions. Due to the lack of replication and the inability to make treatment comparisons, the three genotypes were completely removed from further analyses in the leaf tissue. The other two samples (corresponding to genotype G8 leaves and genotype G16 roots) identified with fewer than five million mapped reads were in different genotypes and tissue types, leaving at least two replicates after removal. Sample removal resulted in 15 and 18 genotypes used in downstream leaf and root tissue analyses, respectively.

**Figure 2:**
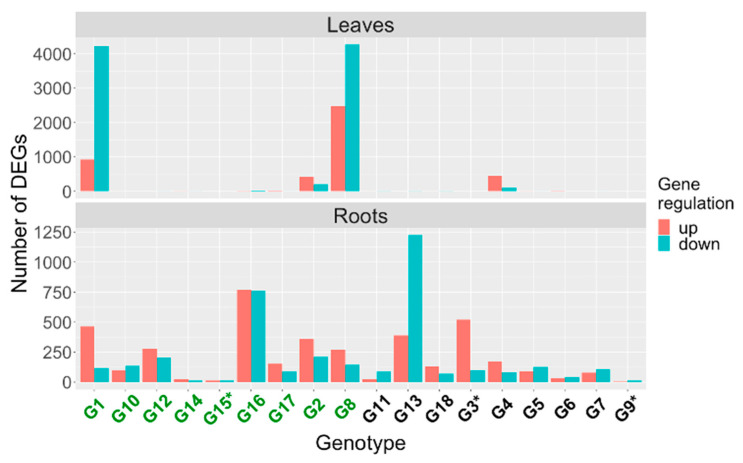
Numbers of differentially expressed genes (DEGs) responding to iron stress across 18 soybean genotypes. Significant DEGs (deficient vs. sufficient; FDR < 0.05) were identified 60 min after iron stress in leaf and root tissue. Genes up-regulated and down-regulated in response to iron stress are shown in red and blue, respectively. Previous hierarchical cluster analysis based on iron stress phenotypic measurements revealed two major clusters of soybean genotypes, iron–efficient and iron–inefficient, shown in green and black, respectively. Three genotypes that were omitted from leaf tissue due to sample removal during sequence processing are indicated with an asterisk.

Following the edgeR workflow, we tested the treatment effect of iron deficiency by comparing the expression of genes in deficient conditions against sufficient conditions within each genotype. The number of differentially expressed genes (DEGs, FDR < 0.05) varied considerably across genotypes in both tissue types (Supplementary Table S1, Supplementary Files S3 and S4). The total number of DEGs ranged from 1 to 6747 in leaves and from 16 to 1611 in roots. Plotting the number of DEGs by tissue type across genotypes clearly demonstrated the variability in numbers of DEGs (Figure 2). Within both the EF and INF groups, we identified distinct patterns of DEG numbers. In the EF group, genotypes G1, G2, and G8 had higher DEG counts in both leaves and roots relative to other genotypes in the group. In genotypes G10, G12, G16, and G17, we identified few DEGs from leaves, but many from roots (<100 in leaves and >200 in roots), and genotype G14 had DEG counts <50 in both leaves and roots. This suggests differences in iron stress responses among the EF group. In the INF group, all genotypes aside from G4 had DEG counts <100 in leaves and a range of DEG counts in the roots.

### 2.3. Comparison of Differentially Expressed Genes between Genotypes

Searching for similar DEGs between individual pairs of genotypes, we compared overlapping DEGs in all pairwise combinations of genotypes (Supplementary Figure S1). The number of overlapping DEGs in a pair of genotypes ranged from 0 to 2837 in leaves and 0 to 135 in the roots. Most comparisons made in the leaf tissue resulted in very few to no overlapping genes. This was not surprising considering fewer than 15 DEGs were found in at least half of the genotypes. However, comparing the three EF genotypes with DEG counts >500, G1 and G8 had 2837 DEGs in common and G1 and G2 had 305 genes in common, whereas G2 and G8 only had 215 DEGs in common. Comparisons in the root tissue resulted in over half of the pairs containing at least 15 overlapping DEGs. Within the EF group, G1 and G2 had the most DEGs in common (57). These results again suggest differences in iron stress responses across genotypes.

### 2.4. Comparisons across Genotypes

#### 2.4.1. Differentially Expressed Genes

In order to identify conserved stress response genes in soybean, we identified DEGs that were most common to all genotypes (Supplementary Table S2, Supplementary File S5). Comparing all genotypes in the leaves, the highest overlap was two DEGs shared by five genotypes, followed by 24 DEGs shared by four genotypes, 192 DEGs shared by three genotypes, and 2992 DEGs shared by two genotypes. Genes that were identified in two or more genotypes were typically found in various combinations between G1, G2, G4, and G8. Three of these genotypes are EF (G1, G2, G8) and one genotype is INF (G4). Most of the overlap between two genotypes occurred between G1 and G8. For the overlap of three genotypes, 97% (187/192) of the genes overlapped with some combination of those four genotypes, and for the overlap of four genotypes, 83% (20/24) of the genes overlapped with those four genotypes. These groupings suggest that some core stress mechanisms may be conserved between these four genotypes.

The two genes, *Glyma.11G190200* and *Glyma.18G104400,* shared across five genotypes, encode a UDP-D-apiose/UPD-D-xylose synthetase and a citrate synthase, respectively. Ahn et al. [23] found silencing UDP-D-apiose/UPD-D-xylose synthetase expression caused changes in plant growth, cell death, and leaf yellowing, similar to IDC. This suggests an increased expression of *Glyma.11G190200* could help to alleviate IDC symptoms. López-Millán et al. [24] demonstrated that iron deficiency caused an increase in the activity of multiple citric acid cycle enzymes, including citrate synthase. An overexpression of the *Malus xiaojinensis Citrate Synthase 1* (*MxCS1*) increases the iron stress tolerance in tobacco [25]. The 24 genes found across four genotypes were involved in growth and various hormone responses. For example, *Glyma.06G102100* is homologous to the *AtEXO* gene, which responds to a brassinosteroid stimulus and is required for cell expansion in leaves [26]. Lisso et al. [27] found that *AtEXO* modifies the sugar responsiveness during seedling growth. Moran Lauter et al. [19] identified eight EXO homologs, including *Glyma.06G102100,* that were repressed in response to 60 min of iron stress in Clark leaves. *Glyma.18G030200* is homologous to the *COI1* gene, which is involved in jasmonate signaling and can inhibit growth and induce defense-related processes [28]. Both *Glyma.06G102100* and *Glyma.18G030200* were down-regulated in response to iron stress in the four genotypes.

In roots, we identified 24 genes found in six or more genotypes, including *Glyma.19G016400,* shared by 17 genotypes. *Glyma.19G016400* is a member of the ATP-binding cassette (ABC) transporter superfamily. This gene family has been associated with many functions of plant development and response, such as the transportation of auxin and secondary metabolites [29]. *Glyma.03G160100*, shared across 14 genotypes, is most homologous to *AtCYP_94_B1*, which is involved in apoplastic barrier formation in the roots and confers salt tolerance [30]. Remarkably, the 24 genes shared across six or more genotypes were significantly overrepresented with the GO terms’ cellular response to potassium ion (GO:0035865) and response to hypoxia (GO:0001666). These genes include two homologs of *AtRAP2.1* (*Glyma.19G026000* and *Glyma.13G060600,* shared in 14 and seven genotypes, respectively) and two homologs of AtADH1 (*Glyma04G,240800* and *Glyma.06G122600,* shared in ten and eight genotypes, respectively). AtRAP2.1 is a negative regulator of abiotic stress responses [31], whereas AtADH1 confers resistance to biotic and abiotic stress [32]. More overlapping genotypes were observed in the roots than in the leaves, suggesting a more uniform recognition and response of the stress in root tissue.

**Figure 3:**
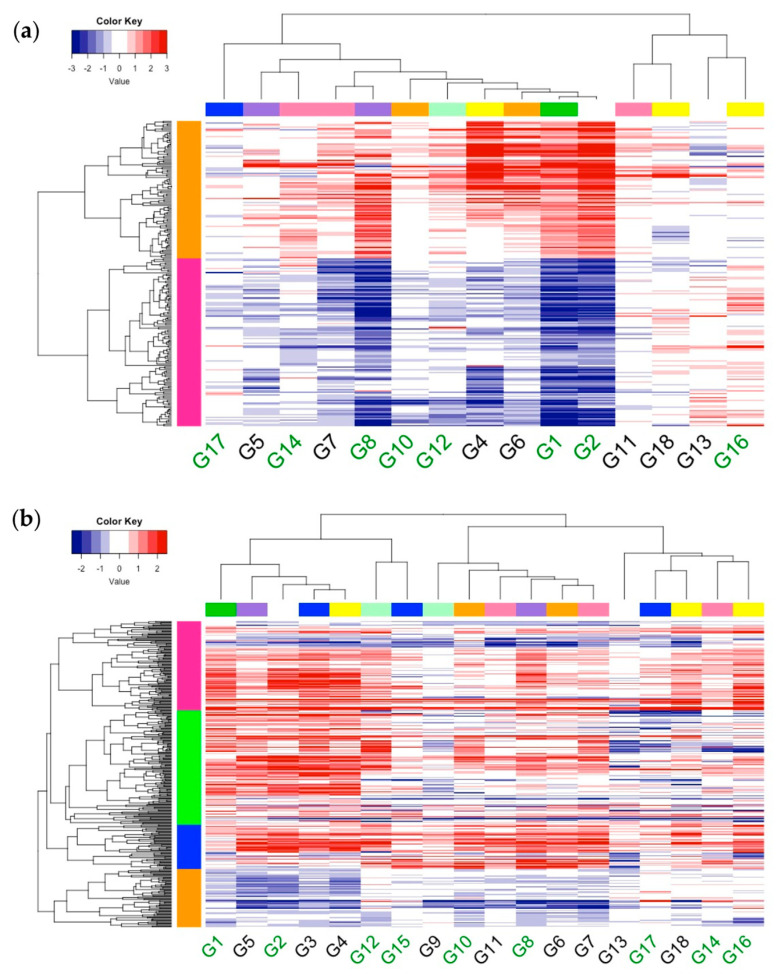
Heatmap of differentially expressed genes across 18 soybean genotypes. Differentially expressed genes (DEGs) in response to 60 min of iron stress response were identified in 18 soybean genotypes. Genes significant in at least three genotypes were identified and then log2 fold–change values were plotted across (**a**) 15 genotypes in leaves and (**b**) 18 genotypes in roots. Three genotypes were omitted from leaf tissue due to sample removal during sequence processing. Haplotypes were identified from sequences within the historic iron deficiency chlorosis (IDC) quantitative trait loci (QTL) on chromosome Gm03. Matching haplotypes are indicated in the color bar above the heatmap. Previous hierarchical cluster analysis based on iron stress phenotypic measurements revealed two major clusters of soybean genotypes, iron efficient and iron inefficient, shown in green and black font, respectively.

To help examine the response of significant DEGs across all genotypes, we generated heatmaps for the 218 and 349 significant DEGs shared by three or more genotypes in leaves and roots, respectively (Figure 3). The hierarchical clustering of the log_2_ fold-change (logFC) allowed us to organize groups of genes and genotypes by similar response profiles. In the leaves, the genotypes clustered into two major clades, but in the roots, the genotypes clustered into three major clades. Interestingly, Clark (G17) was clustered with IsoClark (G18) in roots but not in the leaf tissue. Regardless of the tissue type, there was a combination of EF and INF genotypes within each clade. We found an overlap of genotype groups between tissue types (G13, G16, G18), but were limited in this comparison because of the three genotypes that were removed from the leaf analysis. The clusters found in both tissue types suggest that there are at least two iron stress response mechanisms represented in our mini panel. The hierarchical clustering of the DEGs resulted in two and four major clades in leaves and roots, respectively. Using GO enrichment analysis, we examined the biological processes associated with each DEG clade. In leaves, no GO terms were significantly overrepresented. However, in one nested genotypic clade in leaves (G7, G8, G10, G12, G4, G6, G1, G2), the DEGs were easily grouped by the direction of expression. In the roots, two of the four DEG clades (clade 2 [green] and clade 3 [blue]) contained significantly overrepresented GO terms. Clade 2 was overrepresented with DEGs associated with a response to hypoxia (GO:0001666) and the negative regulation of the ethylene-mediated signaling pathway (GO:0010105). Ethylene is involved in multiple stress signaling, including iron stress [33] and hypoxia [34]. Clade 3 was overrepresented with DEGS associated with a response to hypoxia (GO:0001666) and the sulfolipid biosynthetic process (GO:0046506). The membrane lipid composition can change under stresses, including hypoxia and phosphate starvation [35,36]. Interestingly, Thimm et al. [37] also found expression changes in anaerobic-related genes while using hydroponics but attributed this response to attempted energy production rather than a hypoxia response.

#### 2.4.2. Enriched Biological Process Terms

Our analyses thus far suggest that the genotypes used in this study had largely unique responses to iron stress. Of the 9718 DEGs identified in leaves, 97.7% were only found in one or two genotypes. Similarly, in the roots, 93.8% of DEGs were found in one or two genotypes. However, soybean is a paleopolyploid with multiple genome duplication events in its history [38]. Therefore, it is possible that the gene-specific differences we observed could be due to homeologous genes performing similar functions. To address this issue, we took advantage of GO term enrichment. Homeologous genes should have the same best *Arabidopsis* hit and would be assigned the same GO terms, allowing us to compare biological processes involved in the response to iron stress across genotypes (Supplementary Tables S3 and S4, Supplementary File S6).

In leaves, we found 106 non-repetitive overrepresented GO terms across genotypes. Only two genotypes (G1, G8) had overlapping significant GO terms (corrected *p*-value < 0.05). The lack of DEGs found across genotypes likely contributed to the lack of shared overrepresented GO terms. However, within these two genotypes, were GO terms directly associated with iron (iron–sulfur cluster assembly [GO:0016226] and iron ion homeostasis [GO:0055072]). Additionally, other GO terms related to the defense response and photosynthesis were shared by these two genotypes.

In the root tissue, we found 54 non-repetitive overrepresented GO terms across all genotypes, with up to seven genotypes that were significant for the same overrepresented GO term. Although no GO terms were found with the iron specifically mentioned in the description, multiple GO terms were associated with the signaling or response to stress. The GO term with the highest number of overlapping genotypes (five) was the response to hypoxia (GO:0001666). GO terms that were significant in four genotypes were the hydrogen peroxide metabolic process (GO:0010310), systemic acquired resistance, and the salicylic-acid-mediated signaling pathway (GO:0009862). Hydrogen peroxide (H_2_O_2_) is produced by plants under waterlogged conditions and is associated with stress signaling [39]. Moran Lauter et al. [20] identified GO:0009862 (systemic acquired resistance, salicylic-acid-mediated signaling pathway) as one of the top ten overrepresented GO terms in one cluster of DEGs responding to iron stress in the root.

### 2.5. Comparing Differentially Expressed Genes between Iron Efficiency Groups

In order to understand the relationships between EF and INF genotypes, we compiled a list of total unique DEGs for each tissue type (9720 unique DEGs in leaves and 5633 unique DEGs in roots). We then identified DEGs unique to either EF or INF genotypes and quantified the number of genotypes the DEG considered significantly differentially expressed (Supplementary Table S2, Supplementary Files S7 and S8). Identifying EF- and INF-specific DEGs would help to separate specific iron stress responses from general stress responses.

We identified 9141 and 2848 DEGs unique to EF leaves and roots, respectively (Supplementary File S7). In EF leaves, 6160 were unique to a given genotype, 2859 were shared by two genotypes, 123 were shared by three genotypes, and one was shared by four genotypes. While looking at *Arabidopsis* homologs of DEGs shared by three or more EF genotypes, we found many genes related to stress signaling and tolerance. The only gene unique to four EF genotypes was *Glyma.13G155200*, with the greatest homology to *AtTIL*, a temperature-induced lipocalin. Charron et al. [40] found that an overexpression of *AtTIL* enhanced stress tolerance, delayed flowering, and maintained leaf greenness. Interestingly, this gene was down-regulated in all four EF genotypes under iron-deficient conditions. GO term enrichment analyses of the 9141 DEGs unique to EF leaves identified 93 significant terms, including numerous terms associated with photosynthesis, defense, cell division, iron homeostasis, and the response to iron. In EF roots, 2677 DEGs were unique to a given genotype, 159 DEGs were common to two genotypes, and 12 DEGs were common to three EF genotypes. Among the genes shared by the three EF genotypes in roots were genes responding to stress-related hormones, including: *Glyma.05G222400*, with the greatest homology to *AtACO1*, involved with ethylene biosynthesis and the induction of iron acquisition genes [41]; *Glyma.15G062400*, with the greatest homology to *AtPRB1*, a basic pathogenesis-related protein induced by the defense signaling molecules methyl jasmonate and ethylene [42]; and *Glyma.20G248100*, a bHLH transcription factor (*AtAKS2*) negatively regulated by the stress-related hormone abscisic acid [43]. GO term enrichment analyses of the 2848 DEGs unique to EF roots identified only five significant GO terms: RNA methylation (GO:0001510), nucleosome assembly (GO:0006334), chalcone biosynthesis (GO:0009715), ribosome biogenesis (GO:0042254), and the response to gravity (GO:0009629).

We identified 354 and 2026 DEGs unique to INF leaves and roots, respectively (Supplementary File S8). For INF leaves, 350 DEGs were unique to a given genotype and four DEGs were shared by two genotypes. These encoded a PHD transcription factor, a fatty acid desaturase, and two Kunitz trypsin inhibitors. *AtKT11*, with the greatest homology to *Glyma.08G342100,* regulates plant cell death in plant pathogen interactions [44]. GO enrichment identified 12 significantly overrepresented GO terms, largely associated with hormones (jasmonic acid biosynthesis [GO:0009695], signaling [GO:0009867], response [GO:009753] and metabolism [GO:0009694], ethylene biosynthesis [GO:0009693], and abscisic acid signaling [GO:0009738]) and defense and wounding responses (response to fungus [GO:0009620], regulation of insect defense response [GO:2000068], and response to wounding [GO:0009611]). In INF roots, 1939 DEGs were unique to a given genotype, 77 DEGs were shared by two genotypes, nine DEGs were shared by three genotypes, and one DEG was shared by four and five genotypes. Among the ten genes shared by three or more genotypes, were three genes involved in abiotic stress responses. *Glyma.18g064900*, shared by three genotypes, is most homologous to *AtPTR1* which transports dipeptides as a nitrogen source in the roots [45]. *Glyma.17g162000*, shared by five genotypes, is most homologous to *AtLBD38*, which negatively regulates genes involved in nitrate uptake and assimilation, including members of the peptide transporter family [46]. *Glyma.14g171700*, shared by three genotypes, shares the greatest homology with *AtTPPI*, which regulates stomatal apertures to enhance drought tolerance and water use efficiency [47]. GO term enrichment analyses of the DEGs unique to INF roots identified 16 significant terms associated with defense and wounding responses, including numerous cell wall modifications (secondary cell wall [GO:0009834] and cell wall biogenesis [GO:0009832], cell wall macromolecule metabolism [GO:0044036], lignin [GO:0009809], coumarin [GO:0009805], cellulose [GO:0030244] and glucuronoxylan [GO:0010417] biosynthesis, glucuronoxylan metabolism [GO:0010413], and lignin catabolism [GO:0046274]).

### 2.6. Characterization of DEG Expression Trends within Biological Processes Terms

**Figure 4:**
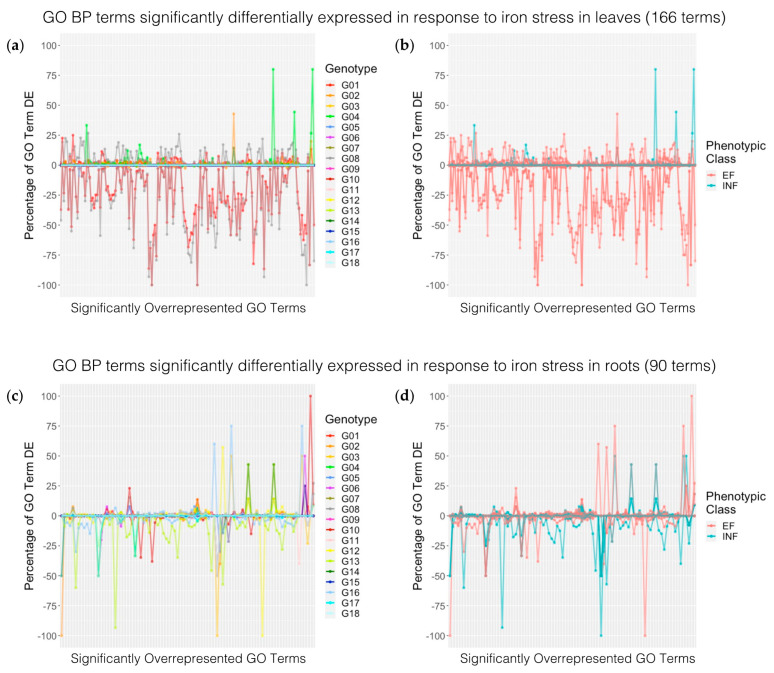
Percentage of differentially expressed genes (DEGs) associated with select gene ontology (GO) terms in leaf tissue of 18 soybean genotypes. GO term enrichment analysis was used on DEGs that were up-regulated or down-regulated in response to 60 min of iron stress in (**a**,**b**) leaf and (**c**,**d**) root tissue of each genotype. DEG numbers for GO terms that were significant in at least one genotype were compiled across genotypes. The percentage of DEGs expressed relative to the total count of that GO term across the genome was calculated for each genotype and plotted with up-regulated genes shown as positive values and down-regulated genes shown as negative values. Genotypes G3, G9, and G15 have values of zero in the leaves due to sample removal during sequence processing. (**a**,**c**) Each genotype is represented by a unique color. (**b**,**d**) Previous hierarchical cluster analysis based on iron stress phenotypic measurements revealed two major clusters of soybean genotypes, iron–efficient and iron–inefficient, shown in red and blue, respectively. Additional data available in Supplementary File S9.

Identifying biological relevance in GO terms in the leaves and roots was challenging due to the lack of statistically significant overrepresentation across genotypes. When reviewing the compiled list of GO terms, many GO terms that were not significantly overrepresented still contained significant DEGs across genotypes. For example, in the GO analysis above, in leaves, 15 GO terms associated with hormone signaling and the defense response were only significant in G4. We found comparable numbers of DEGs associated with these GO terms, but not significant, in G1 and G8. In roots, GO:0009862 (systemic acquired resistance, salicylic-acid-mediated signaling pathway) and GO:0010310 (the regulation of hydrogen peroxide metabolic process) were significantly overrepresented in four genotypes (G2, G3, G4, G5) but have DEGs associated with these GO terms in 17 genotypes. This raised the possibility that our results were impacted by the timing differences between genotypes, and not mechanistic differences. To address this, we needed to visualize how GO terms responded to iron stress across genotypes. We separated DEGs within genotypes based on the direction of expression (induced by iron stress vs. repressed by iron stress). We used GO term enrichment on each DEG list and then compiled a nonredundant list of significant biological process terms. We then used this list to determine how many DEGs were identified in each genotype for each GO term. We identified 168 and 90 significant GO terms in leaves and roots, respectively. We then plotted this data to visualize the expression patterns of different biological process across genotypes in leaves and roots; plots were colored by the genotype and by the iron stress phenotype (Supplementary Figure S2, Supplementary File S9). To adjust for GO terms with small genome counts that would not be easily identified in the plot, we calculated the percentage of genes in the GO term that were significantly differentially expressed relative to the GO term genome count for all GO terms and genotypes (Figure 4, Supplementary File S9). If differences between genotypes were simply timing, all genotypes would have varying peaks under the same GO term. We would expect this pattern for genotypes G1 and G8, which had a number of overlapping DEGs and GO terms in the leaves. If differences between the genotypes were mechanistic, we would see differences in the GO terms associated with different genotypes. For ease of interpretation, we focused on GO terms and genotypes where the number of DEGs was greater than 2% of all genes assigned to the GO term.

For the vast majority of GO terms plotted, the response was largely genotype-specific. One or two genotypes had similar GO term expression patterns, whereas the remaining genotypes had background DEG expression levels that were less than 2%. In the roots, G13 (INF) and G16 (EF) had similar expression trends among 27 GO terms repressed in both genotypes. Interestingly, 17 additional GO terms were induced in G16, but repressed in G13, suggesting differences in timing between the two genotypes, and not different iron stress mechanisms. G2 (EF) also shared six repressed GO terms with G13 (INF), which were induced in G16 (EF). These GO terms included four signaling hormones involved in the stress response: ethylene (GO:0009873), salicylic (GO:0009862 and GO:0009863), and jasmonic acid (GO:0009753) [48,49]. In the leaves, genotypes G1 (EF) and G8 (EF) shared similar expression trends for 135 GO terms among repressed DEGs and 54 GO terms among induced DEGs. Genotypes G2 (EF) also shared 28 GO terms with genotypes G1 and G8, but only among induced DEGs (expression > 2%). This suggests that genotypes are using different strategies to cope with iron stress.

Comparing phenotypic groups in leaves, we found three EF genotypes (G1, G2, G8) with induced gene expression and two EF genotypes with repressed gene expression (G1, G8). However, in leaves, only one INF genotype responded (G4). Remarkably, G4 only induced gene expression (>2%). If we examine the 168 GO terms identified in leaves, 141 are specific to EF groups (INF expression < 2%), 3 are specific to INF genotypes (EF expression < 2%), and 24 are common to the EF and INF genotype. The three GO terms unique to INF (G4) included the regulation of sequence-specific DNA binding transcription factor activity (GO:0051090), regulation of transcription from RNA polymerase II promoter in response to oxidative stress (GO:0043619), and regulation of the defense response to insects (GO:2000068). Eight GO terms involved in jasmonic acid processes and defense response were expressed in G1 (EF), G4 (INF), and G8 (EF). EF-specific terms were associated with a range of processes, including photosynthesis, methylation, defense, iron homeostasis, the regulation of transcription and translation, and growth. A lack of expression of other INF genotypes in the leaves suggests that either INF genotypes are not able to respond to iron stress in the leaves, or that INF roots are unable to signal iron stress to the leaves, which could be an important distinction between EF and INF genotypes. Additionally, the range of responses found in EF leaves suggests a cascade of iron stress responses, whereas the response of INF leaves seems to be a more general defense response.

We saw induced and repressed GO terms in the root for EF and INF genotypes. If we examine each GO term in the root, 23 were specific to EF groups (expression < 2% across INF genotypes), 3 are specific to INF groups (expression < 2% across EF genotypes), and 64 could be found in EF and INF genotypes. INF-specific GO terms were associated with nucleotide–sugar metabolism (GO:0009225), the response to fructose (GO:0009750), and chaperone-mediated protein folding (GO:0061077). EF-specific terms were associated with stress, defense, DNA replication, cell division, and methylation. Interestingly, two genotypes (G14, G15) had little to no overlap of GO terms in roots, suggesting distinct iron stress responses.

### 2.7. Characterization of Differentially Expressed Transcription Factors

**Figure 5:**
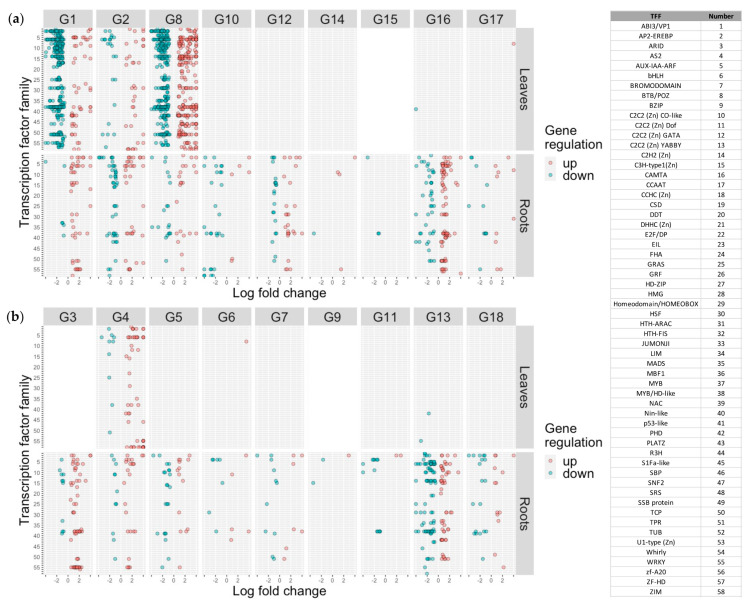
Expression values of differentially expressed transcription factors of 18 soybean genotypes. Differentially expressed genes were identified in leaf and root tissue in response to 60 min of iron stress. Transcription factors were identified in each DEG list and then plotted by transcription factor family using the log_2_ fold–change of the stress response. Genes considered up- or down-regulated are shown in red or blue, respectively. Genotypes are divided by iron efficiency ((**a**) iron–efficient, (**b**) iron–inefficient) based on hierarchical clustering of phenotypic data. Genotypes G3, G9, and G15 contain blank cells in leaf tissue due to sample removal during sequence processing.

In order to identify regulators of potential pathways of interest, we identified DEGs annotated as transcription factors (Supplementary Table S5, Supplementary File S5). Log_2_ fold-change values of differentially expressed transcription factors (TFs) grouped by the transcription factor family (TFF) were plotted for each genotype × tissue type (Figure 5). In leaves, we identified 897 TFs belonging to 56 TFFs. Most (92%) of the TFs were unique to EF genotypes, 43 TFs (5%) were unique to INF genotypes, and only 25 (3%) of TFs were found in at least one genotype of each phenotypic group. Of the 56 TFFs identified in leaves, 16 TFFs were found in both phenotypic groups, 40 TFFs were unique to EF in leaves, and no TFFs were unique to INF in leaves. In roots, we identified 569 TFs belonging to 49 TFFs. Almost half of the TFs (47%) were unique to EF, fewer TFs were unique to INF (36%), and only 17% of TFs were found in at least one of each phenotypic group. Similar to leaves, all TFFs identified in INF genotypes were identified in EF genotypes, whereas 12 TFFs were unique to EF in roots. Interestingly, 71% and 78% of the TFs were unique to a single genotype in the leaves and roots, respectively. An overlap of TFF between phenotypic groups could suggest similar target pathways for a general stress response, with additional target pathways that distinguish the EF genotypes.

The expression patterns in TFs were similar to the expression patterns of total DEGs. We found that EF genotypes (G1, G2, G8) had relatively strong numbers in the leaves and roots. Most of the other EF genotypes (G10, G12, G16, G17) had consistent numbers of TFs in the roots, but little to no TFs in the leaves. The remaining EF genotypes (G14, G15) had few TFs in either the leaves or roots. Most TFs for INF genotypes were identified in the roots. Only one INF genotype (G4) had a noticeable number of TFs in the leaves. Two INF genotypes (G6 and G13) had only one and two TFs identified in the leaves, respectively. Higher numbers of TFs observed in the leaves of EF genotypes suggests a more active response to the iron stress.

We performed TF enrichment analyses to identify overrepresented transcription factor families (TFF; Supplementary Figures S3 and S4, Supplementary File S10). Surprisingly, we did not find many significantly overrepresented TFFs, suggesting a range of processes that are targeted during the stress response. In leaves, we identified four overrepresented TFFs: C2C2 [Zn] CO-like (G1 and G8), MYB/HD-like (G1), SBP (G8), and ZIM (G2 and G4). In roots, we identified three overrepresented TFFs: AS2 (G6 and G11), WRKY (G3), and ZIM (G10). Most overrepresented TFFs in leaves overrepresented TFs belonged to EF genotypes, and, in roots, overrepresented TFs belonged mostly to INF genotypes.

### 2.8. Characterization of Differentially Expressed Genes across IDC QTL

The increasing body of IDC literature has identified numerous regions across the soybean genome that are associated with the iron stress response. Assefa et al. [12] identified 69 regions of interest as a result of a GWAS using 460 diverse lines, including the lines in our mini-panel. The 69 regions correspond to 278 candidate genes, some supported with previous RNA-seq data from our group, which were restricted to genotypes Clark (G17, [19,20]) and IsoClark (G18, [50]). Cross referencing our lists of overlapping DEGs in both tissue types (Supplementary File S5), we identified 67 candidate DEGs corresponding to 43 of these 69 regions. A total of 49 genes were unique to leaves, 12 genes were unique to roots, and 6 genes were common to both tissue types. Some DEGs are specific for iron-stress responses, whereas others are for more general stress responses. For example, *Glyma.02G075100* is homologous to *AtSUC2*, a sucrose transporter gene that increases iron deficiency tolerance when overexpressed in *Arabidopsis* [51]. Moran Lauter et al. [19] found that *Glyma.16g157100*, which is homeologous to *Glyma.02G075100*, is induced in Clark (G17) leaves six hours after iron stress. *Glyma.05G000300* encodes an iron–sulfur cluster containing ferredoxin–thioredoxin reductase enzymes and was identified as a candidate gene for IDC tolerance by Butenhoff [52] using the Fiskeby III x Mandarin (Ottawa) mapping population. *Glyma.06G056400,* homologous to *AT2G26330,* encodes a leucine-rich repeat receptor-like kinase. Shanmugam et al. [53] overexpressed a truncated dominant-negative *Arabidopsis* ERECTA gene in soybean and observed a decrease in plant development and an increase in stress response. *Glyma.14G031700* is homologous to *AtWDR26*, a WD-40 repeat containing protein. An overexpression of *AtWDR26* induced gene expression across a range of processes, including hormone, light, and abiotic stress [54]. Among the six genes common to both tissue types was *Glyma.03G144500*, which is homologous to the *FAD2* gene. Yuan et al. [55] found that *FAD2* is involved in the plant response to phytohormones and abiotic stress.

Based on the location of the Gm03 QTL defined by Assefa et al. [12], we further explored DEGs within this region. We identified 10 DEGs unique to leaves, four unique to roots, and one gene significant in both tissue types. Surprisingly, DEGs that were located within the Gm03 QTL were only identified in five genotypes, not including Clark or IsoClark, between both tissue types. In leaves, only G1 and G8 had DEGs identified in the Gm03 QTL, two of which were significant in both genotypes (*Glyma.03G128900* and *Glyma.03G130300*). Assefa el al. [12] identified *Glyma.03G128900*, homologous to *AtLCY*, as a high priority candidate gene in region one of the Gm03 QTL. The transformation of β-lycopene cyclase genes from *Salicornia europaea* L. into both *Arabidopsis* and tobacco increased carotenoid retention and improved oxidative and salt stress tolerance [56]. O’Rourke et al. [57] also identified *Glyma.03G130300* as differentially expressed in leaves 24 h after iron stress and in roots after multiple exposures to iron and phosphate stress. Another gene of interest was *Glyma.03G128300*, which is homologous to the glutamate synthase, *AtGLU1*. Knock-down *Arabidopsis* mutants showed large transcriptional changes to various pathways, including photosynthesis and stress response [58], while Cui et al. [59] found *AtGLU1* to be involved in iron homeostasis. In roots, three genotypes (G2, G13, G16) had DEGs in the Gm03 QTL. Remarkably, two genes (*Glyma.03G131200* and *Glyma.03G131400*) were annotated as the same protein, but were differentially expressed in different genotypes (G13 and G2, respectively). Both genes were homologous with members of the 2-oxoglutarate (2OG) and Fe(II)-dependent oxygenase superfamily. These non-heme enzymes utilize ferrous iron as a co-factor, catalyze a wide range of reactions, and are potentially involved in sensing the iron status [60,61]. Moran Lauter et al. [19] identified *Glyma.07g150900*, also a member of the 2OG-Fe(II)-dependent oxygenase superfamily, as differentially expressed in Clark (G17) roots one hour after iron stress. *Glyma.03G130200* was identified in leaves (G1) and roots (G16) and is homologous with a strictosidine synthase-like (SSL) protein. Sohani et al. [62] demonstrated that members of the SSL gene family are involved in plant defense mechanisms.

Zhang et al. [63] used image analysis and machine learning to rate iron deficiency chlorosis. In a GWAS using the image analysis output, they identified seven QTL associated with iron deficiency across the genome. Within an 847 kb region on Gm03 (overlapping the historic IDC QTL on Gm03), they identified seven candidate genes. One of the seven candidate genes located on Gm03 (*Glyma.03G128300*) was identified in the leaves (G8) and two (*Glyma.03G131200* and *Glyma.03G131400*) were identified in the roots (G13, G2). All three genes on Gm03 were highlighted in the previous paragraph. An additional 2OG-Fe(II)-dependent oxygenase (*Glyma.18G111000*) 41.4 kb downstream from another variant found on Gm18 was also identified in the leaves (G8). These findings highlight the utility of leveraging early gene expression studies with GWAS field studies to identify candidate genes controlling agronomically important traits.

### 2.9. Single Linkage Clustering

We used single linkage clustering to group iron-stress-responsive DEGs (13,980) by shared sequence homology (TBLASTX, E < 10–20) or individual genes shared across multiple genotypes, tissues, or expression patterns. Of the 13,980 unique DEGs identified in our experiment, 12,138 DEGs clustered into 2922 clusters. Clusters ranged in size from one DEG to 2136 DEGs, and represented up to 18 genotypes (Supplementary Figure S5). Of the 2922 clusters, 1763 and 50 were specific to EF and INF genotypes, respectively. On average, EF clusters contained 2.28 DEGs (STD = 1.9), whereas INF clusters contained 2.02 DEGS (STD = 1.37). Similarly, EF clusters represented 2.28 genotypes (STD = 0.65), whereas INF clusters represented 1.48 genotypes (STD = 0.58). The limited number of genotypes found on average in each cluster again suggests that most genotypes respond very differently to iron stress.

## 3. Discussion

Soybean is a major cash crop grown in the Midwest; due to various soil properties, soybeans grown in this geographic region of the United States have a higher chance of developing the nutrient stress, iron deficiency chlorosis. Although many studies have contributed to the current knowledge of the molecular response of soybean to IDC, no study has investigated the variation of the molecular response across a wide breadth of the germplasm collection. Similarly, studies in model species have largely focused on one or two main genotypes. Therefore, we sought to compare the early responses to IDC across a diverse panel of soybean genotypes to identify both differences in the stress response across genotypes and novel IDC tolerance mechanisms to exploit in the future.

### 3.1. Soybean Responds Rapidly to Iron Stress

Plants have the ability to rapidly respond to changes in environmental conditions in scales of seconds and minutes [64]. Buckhout et al. [65] examined the early iron stress response of *Arabidopsis* in a time-course analysis by examining differential expression at 0, 0.5, 1, 6, and 24 h after iron stress. While their analysis shows differential expression at all timepoints, they concluded the initiation of the iron deficiency stress response to be sometime between 1 and 6 h after stress. They interpreted that DEGs identified at the first three timepoints were not iron-specific because they were only identified at a single timepoint. In soybean, Atencio et al. [21] compared Clark (G17) iron stress responses observed by Moran Lauter et al. (30, 60, 120 min after iron stress) [20] and O’Rourke et al. (24 h after iron stress) [57] to their own study (two and ten days after iron stress). Of the 9102 and 15,881 DEGs unique to leaves and roots, respectively, approximately 60% were unique to a single time point. While the majority of genes were specific to a given timepoint, they included the hallmarks of the Clark (G17) iron stress response: genes involved iron homeostasis, defense response, and DNA replication/methylation [18,19,20,57]. In this study, 67% and 82% of DEGs identified in leaves and roots, respectively, were unique to a single genotype. This suggests that the majority of soybean genotypes in our panel, and not just Clark (G17), are able to recognize and respond to iron stress within 60 min.

Khan et al. [66] examined expression levels of the canonical *Arabidopsis* genes *OPT3*, *FIT,* and *IRT1* and detected expression at 4, 8, and 12 h after iron stress, respectively. Since *OPT3* was detected earliest in the leaves, and *FIT* and *IRT1* were detected later in the roots, they suggested that leaves sense changes in iron availability more quickly than roots. In contrast, Moran Lauter et al. [20] found higher numbers of DEGs in Clark (G17) roots than in the leaves at the earliest timepoint of 30 min after stress, suggesting that roots respond more quickly than leaves to iron stress in soybean. Examining GO terms across timepoints and tissues revealed that the same GO terms were affected, first in the roots, then in the leaves, suggesting a root-to-shoot signal in soybean. Here, we identified varying numbers of DEGs in the leaf and root tissue across 18 soybean genotypes. For the majority of genotypes, more DEGs were identified in the roots than the leaves, supporting early root-to-shoot signaling in soybean. Only four genotypes had more DEGs identified in the leaves than the roots. Interestingly, three of the four genotypes with more DEGs in leaves than roots were EF (G1, G2, G8), suggesting that these lines respond faster than Clark (G17), where leaf expression was just beginning at 60 min [20]. Future gene expression studies using a variety of soybean genotypes would benefit by including multiple timepoints to enhance our understanding of the timing and movement of the stress signal across genotypes.

### 3.2. Diversity of Iron Stress Responses Found within the Soybean Germplasm Collection

Many studies across plant species have utilized RNA sequencing (RNA-seq) to identify genes, pathways, and networks that are triggered in response to stress. Due to their expense, early RNA-seq studies focused on one or two genotypes with contrasting stress responses. Recently, studies have begun to increase the number and diversity of genotypes used with RNA-seq to identify novel genes and pathways associated with a trait or stress response [67,68,69,70]. Stein and Waters [71] and Waters et al. [72] compared the iron stress response from the root and rosette tissues of five *Arabidopsis* ecotypes. Their conclusions highlighted a small handful of ‘core’ iron stress response genes overlapping between ecotypes. The differentially expressed genes not shared between ecotypes were thought to represent genotype x environment interactions, and not primary Fe-responsive genes. However, genotype by environment (GxE) interactions are critically important for crop improvement. A recent review by Cooper and Messina [73] highlighted the importance of leveraging cross disciplinary approaches in order to both understand GxE interactions and accelerate crop improvement. Within soybean, traditional genetic studies demonstrate the existence of multiple iron stress tolerance mechanisms. Lin et al. [9] used two mapping populations to study the IDC response in soybean. One population (Pride B216 × A15) found a minor effect QTL on six linkage groups, and the other population (Anoka × A7) found a single major effect QTL, suggesting that there are at least two distinct mechanisms that control the IDC response in soybean. Butenhoff [52] and Merry et al. [74] used the same mapping population (Fiskeby III × Mandarin [Ottawa]) and identified QTL on three chromosomes. Both studies found a QTL on Gm05, and Merry et al. [74] additionally identified QTL on Gm03 (same as previously identified IDC QTL [9,15]) and Gm06. Merry et al. [74] suggested that the QTL on Gm05 contains significant variation for future breeding efforts due to low minor allele frequencies of the iron-inefficient alleles on Gm03 and Gm06 among elite breeding lines. In this study, we identified DEGs in the same regions on all three chromosomes defined by Merry et al. [74] and for 43 GWAS regions identified by Assefa et al. [12], suggesting that these regions, identified in different genotypes and studies, contain important genes for iron stress responses in soybean. In Figure 1, the EF genotypes clearly cluster by the phenotype. In Figure 4, the EF genotypes have largely distinct expression patterns and mechanisms from themselves and INF genotypes. We believe that these differences represent novel resources to enhance the iron stress tolerance in soybean.

### 3.3. Identifying Targets for Future Analyses

We have cross referenced the DEGs identified in this study with Clark gene expression studies conducted by Moran Lauter et al. [19], Moran Lauter et al. [20], Atencio et al. [21], and O’Rourke et al. [57]. Of the 9718 and 5632 unique DEGs identified in the leaves and roots of this study, 5491 (56.5%) and 3493 (62.0%) were identified in at least one tissue sample of the Clark studies above (Supplementary Tables S6–S8). Remarkably, 4227 and 2140 DEGs from leaves and roots remain unique to this study. Of these, 1247 DEGs from leaves and 289 DEGs from roots were identified in at least one other genotype. In Supplementary File S5, we provide the DEGs identified in this study and the corresponding genotype information (total Genotypes, EF Genotypes and IN Genotypes). We have cross referenced the DEGs with the previously identified Clark iron-stress-responsive DEGs, we have identified DEGs falling within GWAS QTL identified by Assefa et al. [12], and we have provided multiple annotation sources. It is our hope that we and others can use this information to prioritize candidate genes for future functional characterization in soybean and other crop species.

**Figure 6:**
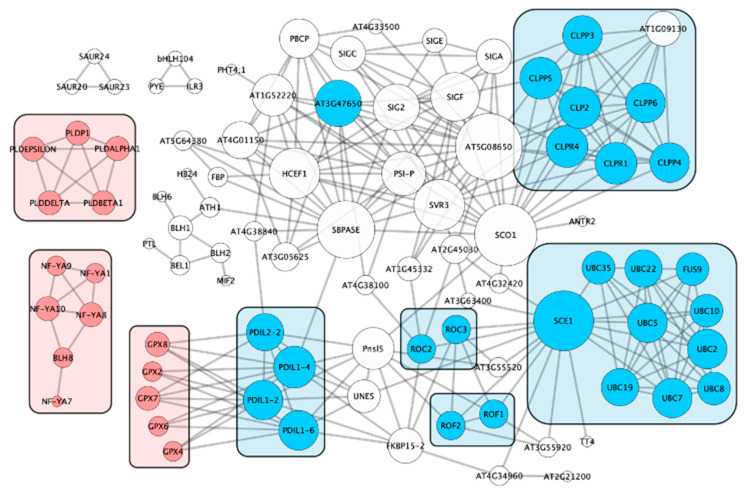
Interactions of Arabidopsis homologs of differentially expressed soybean genes. Differentially expressed genes (DEGs) were identified across 18 soybean genotypes and two tissue types (leaves and roots) 60 min after iron stress. Single linkage clustering was used to identify DEGs with shared sequence homology. Previous hierarchical cluster analysis based on iron stress phenotypic measurements revealed two major clusters of soybean genotypes, iron–efficient (EF) and iron–inefficient (INF). Arabidopsis homologs were identified for the 25 largest EF–specific clusters and used with STRING (version 11.5) to identify protein interactions of the Arabidopsis homologs. Cytoscape (version 3.7.2) was used to visualize the interaction network of proteins with at least one interaction. Six soybean clusters, highlighted in blue, were associated with protein regulation, including quality control (cluster 606), folding (clusters 258, 334, 406, and 842), and modification (cluster 392). Three soybean clusters, highlighted in red, were associated with endoplasmic reticulum (ER) stress (clusters 218, 556, and 689).

To demonstrate novel ways that these data sets could be leveraged, we focused on the 25 largest EF-specific clusters identified with single linkage clustering (Supplementary File S11). In order to investigate if the EF clusters might interact, we took the 308 DEGs corresponding to the 25 EF-specific clusters and identified their best Arabidopsis homolog (120 total unique proteins). We then used STRING (ver. 11.5, [75]) to visualize interactions among the clusters (Figure 6). 

The majority of clusters were associated with a single large network. Within the network, six clusters could be directly associated with protein regulation, including quality control (cluster 606), folding (clusters 258, 334, 406, 842) and modification (cluster 392). CLP proteases (cluster 606) degrade misfolded proteins [76]. Peptidyl-prolyl cis-trans isomerases (clusters 258 and 406, [77]), protein disulfide isomerases (Cluster 334, [78]), HSP40s (see review [79]), and other chaperones (cluster 842, [80]) catalyze protein folding in the endoplasmic reticulum (ER). Ubiquitination targets proteins for degradation [81], whereas SUMOylation can regulate protein function (cluster 392, [82]). In plants, abiotic and biotic stress can result in misfolded proteins, which accumulate in the ER and cause ER stress, toxicity, and programmed cell death (see review [83]). In order to maintain ER homeostasis, cells activate the unfolded protein response, upregulating genes involved in preserving the protein quality and quantity [84]. While genes involved in the unfolded protein response were not statistically overrepresented in our study, they were significantly overrepresented in Clark roots at 30 min after iron stress [20]. Since this study focused on 60 min after iron stress, it suggests that we are observing downstream stages of the unfolded protein response, and not the initiation. The analysis of overrepresented terms within STRING supports this hypothesis: DEGs associated with protein quality control (GO:0006515) and protein folding (GO:0006457) are significantly overrepresented.

We also identified other clusters within the STRING network that could be associated with ER stress. In plants, phospholipase D (cluster 218), is associated with tolerance to osmotic and temperature stress, plant pathogen defense, phosphate and nitrogen deficiencies, and heat stress memory (see review [85,86]). However, recent work in mammalian systems has demonstrated that the inhibition of phospholipase D results in ER stress [87]. Similarly, ER stress activates glutathione-related enzymes, including glutathione peroxidases (Cluster 556, [88]). NF-Y transcription factors (Cluster 689) form a transcriptional complex with BZIP60 to bind an ER stress response element located in the promoter of genes involved in unfolded protein responses (see review [83]).

The unfolded protein response is broadly conserved across eukaryotes [89] and responds to a variety of abiotic and biotic stresses, including heat, cold, salinity, drought, flooding, high light, heavy metals, and pathogens [90]. Stress signaling is importance for striking a balance between survival and continued growth and development. To our knowledge, the unfolded protein response has not been tied to iron stress responses in any plant species. Remarkably, while the response is well conserved, in our study, it is largely limited to iron-efficient genotypes G1 and G8. Of the DEGs associated directly or indirectly with the unfolded protein response above, 83% came from G1 and G8. This suggests that these genotypes are able to exploit the unfolded protein response through novel signaling mechanisms. This is just one example of how the data from this study can be exploited for improving soybean iron stress responses.

## 4. Conclusions

Utilizing a variety of genotypes, with a range of phenotypic responses to IDC, can help to improve our understanding of the diverse iron stress responses found in the soybean germplasm. We used RNA-seq to analyze patterns and early changes of gene expression across 18 soybean genotypes in response to iron stress. Changes in gene expression profiles across the genotypes at 60 min after stress demonstrate that the rapid response to iron stress is not limited to the Clark genotype. Variation in the differentially expressed genes and biological processes identified in the early response demonstrated that multiple response mechanisms and potential differences in the response time to iron stress exist in soybean germplasm. While this study highlights differences between genotypes at 60 min after stress, future work would benefit with the inclusion of additional timepoint(s) to analyze changes in signaling pathways and to develop an iron stress response curve for multiple genotypes over time. Ultimately, this study reveals the utility of expanding gene expression studies to include a variety of genotypes.

## 5. Materials and Methods

### 5.1. Phenotypic Clustering

Visual iron deficiency chlorosis (IDC) ratings and soil plant analysis development (SPAD; Spectrum Technologies, Aurora, IL, USA) measurements were collected at multiple growth stages from plants grown in the field in 2014, and in the field and hydroponics in 2015 as described by Assefa et al. [12]. The ‘stats’ package in RStudio [91,92] was used to calculate a distance matrix using the Euclidean method, and then genotypes were clustered using Ward’s method. The same package was used for the principal components analysis (PCA).

### 5.2. Plant Materials

Eighteen diverse plant introduction (PI) lines were selected from a genome wide association study (GWAS) panel used by Assefa et al. [12]. The selected lines comprised eight haplotypes, corresponding to four linkage blocks spread across a historical IDC quantitative trait loci (QTL) on soybean chromosome Gm03 (Supplementary File S1). Based on the field and hydroponic phenotypic data reported by Assefa et al. [12] genotypes from the same haplotype but with contrasting severity ratings in response to IDC were selected. The goal of including contrasting severity ratings was to assess the impact of other genomic locations on IDC tolerance. Two near-isogenic lines (NILs) historically used to study IDC responses, iron-efficient Clark (PI 548533), and iron-inefficient IsoClark (PI 547430), were included to serve as internal controls. Additional distinguishing characteristics that were maintained for our records were the country of origin and maturity group.

### 5.3. Tissue Collection

Seeds of each genotype were germinated on paper in a growth chamber set at 24 °C with a 14 h day length. After seven days in the growth chamber, seedlings were transferred to eight hydroponic buckets, where each bucket contained one seedling of each genotype. All buckets were setup using an iron-sufficient (100 μM Fe[NO_3_]_3_•9H_2_O) hydroponic system in a single growth chamber, as outlined by O’Rourke et al. [17], with nutrient solutions described by Chaney et al. [93] adjusted for 10 L buckets. Hydroponic systems using these nutrient solutions have been used to identify and validate soybean iron stress tolerance QTL found in field conditions [12,94]. After nine days of growth in the hydroponic solution (16 days after germination), seedlings grown in the same bucket were transferred to a new 10 L bucket with either iron-sufficient or iron-deficient conditions (100 μM Fe[NO_3_]_3_•9H_2_O and 50 μM Fe[NO_3_]_3_•9H_2_O, respectively), resulting in four biological replicates of each genotype in each iron condition. During transfer, the group of seedlings was carefully rinsed in solution of the same iron condition as the destination bucket. Moran Lauter et al. [20] observed a shift in root-to-shoot differential gene expression in Clark over the course of 30–120 min, with an inflection point at 60 min after the onset of iron stress. Therefore, we decided to collect tissue samples 60 min after iron stress; this would allow us to capture stress responses in both roots and leaves from genotypes with faster and slower responses relative to Clark. Sixty minutes after transferring the seedlings to new iron conditions, leaflet tissue from the first trifoliolate and whole root tissue were harvested, frozen in liquid nitrogen, and then maintained at −80 °C. All tissue was collected and stored in individual 50 mL Falcon^®^ tubes (Thermo Fisher Scientific, Waltham, MA, USA). Three biological replicates were collected from each genotype and iron condition. The remaining biological replicate for each iron condition was grown for two more weeks to validate phenotypic responses, especially of Clark and IsoClark under iron-deficient conditions (data not shown).

### 5.4. RNA Isolation and Sequencing

Frozen tissue was crushed with an inverted pestle in the 50 mL Falcon^®^ tubes used in tissue collection. One full microspatula scoop (approximately 100 mg) of crushed tissue was transferred to a 2 mL Safe-Lock™ microcentrifuge tube (Eppendorf, Hamburg, Germany), and then ground with a 5 mm stainless steel bead for one minute at 30 Hz using the Qiagen Tissuelyser II (Qiagen, Germantown, MD, USA). RNA was extracted following the RNeasy^®^ Plant Mini Kit protocol. Extracted RNA was DNase treated in 50 μL reactions using the Ambion^®^ TURBO DNA-free™ Kit (Thermo Fisher Scientific, Waltham, MA, USA) and further purified using an RNeasy^®^ MinElute^®^ Cleanup Kit (Qiagen, Germantown, MD, USA). Final RNA concentration and quality was measured using a NanoDrop™ 1000 Spectrophotometer (Thermo Fisher Scientific, Waltham, MA, USA).

RNA samples were sequenced at the Iowa State University DNA Facility. Prior to sequencing, the DNA facility validated the quality of each RNA sample using an Agilent^®^ 2100 Bioanalyzer™ (Agilent^®^, Santa Clara, CA, USA). After quality confirmation, sequences were generated on the Illumina HiSeq 2500 platform (Illumina Inc., San Diego, CA, USA) using normal output mode with 150 base pair, single-end sequencing. A total of 216 samples were run on 19 lanes across three eight-lane flow cells (two full and one partial). Each lane was assigned one rep of six genotypes from one tissue type from both iron conditions (sufficient and deficient).

### 5.5. Identification of Differentially Expressed Genes in Response to Iron Stress

Sequencing adaptors were removed using the program Scythe (version 0.981, [95]), the first 15 bases were removed using the program fastx_trimmer (version 0.0.14, http://hannonlab.cshl.edu/fastx_toolkit, released on 5 January 2014), and bases with quality scores below 20 were removed using the program Sickle (version 1.2, [96]). Cleaned fastq files were sorted and mapped to the soybean reference genome (*Glycine max* Wm82.a2.v1, Phytozome version 12) using TopHat2 (version 2.1.1, [97]). SAMtools (version 1.6, [98]) was used to filter for and reliably sort mapping reads. The resulting binary alignment/map (BAM) files were used for downstream differential expression analyses. BAM files and raw fastq files generated by this study were deposited in the National Center for Biotechnology Short Read Archive (NCBI SRA BioProject accession PRJNA706999). 

RStudio [91,92] was used for statistical analyses. Samples with fewer than five million mapped reads were removed from further analysis. If two of the three reps within iron treatment and tissue type contained fewer than five million mapped reads, the genotype within that tissue type was removed from further analysis. The “edgeR” package [99] was used to identify differentially expressed genes (DEGs). Genes with counts per million (cpm) of one or more (cpm ≥ 1) in at least three samples were considered expressed and used for further analyses. Library sizes were normalized across all samples within tissue type using the trimmed mean of M-values (TMM) method [100]. We fit a negative binomial generalized log-linear model to the normalized count data with genotype x iron condition groups as the factor in our design matrix. Individual contrast statements were made between iron conditions (deficient versus sufficient) of a given genotype within tissue type. The likelihood ratio test was used with each contrast to test for differential expression of the treatment effect by genotype. Genes with a false discovery rate of less than 0.05 (FDR < 0.05) were considered differentially expressed.

### 5.6. Gene Annotation

All DEGs were annotated using the *Glycine max* Wm82.a2.v1 ‘Gene Annotation Lookup’ under the SoyBase Toolbox tab (https://soybase.org/genomeannotation/, released June 2015) [101]). This annotation tool returns the BLASTP (E < 10^−6^, [102]) results of the top hit for the Uniref100 database [103], the most descriptive hit from the Uniref100 database, and the top *Arabidopsis* hit from the TAIR10 database [104]. Additionally, gene ontology descriptions and IDs for biological processes, molecular function, and cellular components associated with the top *Arabidopsis* hit are included. To identify transcription factors within our DEGs, we took advantage of the SoyDB transcription factor database [105]. The SoyBase ‘Gene Model Correspondence Lookup’ (https://soybase.org/correspondence/, released June 2015) was used to update transcription factors to *Glycine max Wm82.a2.v1* gene calls.

### 5.7. Identification of Overrepresented Gene Ontology (GO) Terms and Transcription Factors

A Fisher’s exact test [106] with a Bonferroni correction (corrected *p*-value < 0.05, [107]) was used to test for enriched GO terms associated with a DEG list of interest compared to all genes in the soybean genome. The same approach was used to identify significantly overrepresented transcription factor families.

### 5.8. Single Linkage Clustering

To identify gene families that might play a role in iron stress adaptions and single genes important across multiple genotypes, we used a single linkage clustering approach, as described by Graham et al. [108] and O’Rourke et al. [17]. Custom perl scripts were used to generate a FASTA file of DEGs for each genotype × treatment × expression combination. To each DEG identifier, we added genotype and tissue information (L01-L18 [leaves] or R01-R18 [roots]) and direction of expression (+ induced by iron stress,—repressed by iron stress). For example, Glyma.10G027100 became LG01+_Glyma.10G027100. Note that a particular DEG could be found in different genotype FASTA files, but with different genotype, tissue, and expression information added to the name. Genotype FASTA files were then combined into a single FASTA file, generating a database of iron-stress-responsive genes. TBLASX [102] was used to compare the database against itself using an E-value cutoff of E < 10^−20^. Perl scripts generated by Graham et al. [108] were then used to assign homologous sequences to a cluster.

## Data Availability

Raw fastq and cleaned and sorted BAM files generated during the current study are available in the National Center for Biotechnology Small Reads Archive (NCBI SRA, http://ncbi.nlm.nih.gov/sra, accessed September 2021) under BioProject PRJNA706999. Datasets generated or analyzed during this study are included in this published article and its additional files.

## Supplementary Materials

The following are available online at https://www.mdpi.com/article/10.3390/ijms222111643/s1.

## Abbreviations

CPM	count per million
DEGs	differentially expressed genes
EF	iron-efficient
FDR	false discovery rate
GO	gene ontology
GWAS	genome-wide association study
IDC	iron deficiency chlorosis
INF	iron-inefficient
logFC	log_2_ fold-change
NCBI SRA	National Center for Biotechnology Small Reads Archive
PI	plant introduction line
QTL	quantitative trait loci
ROS	reactive oxygen species
RNA-seq	RNA sequencing
SNP	single nucleotide polymorphism
SPAD	soil plant analysis development
TAIR10	The Arabidopsis Information Resource version 10
TF	transcription factor
TFF	transcription factor family
TMM	trimmed mean of M-values
