# Supplementary material for: Comparing Early Transcriptomic Responses of 18 Soybean (Glycine max) Genotypes to Iron Stress
Source: Int J Mol Sci. 2021 Oct 28;22(21):11643. doi: 10.3390/ijms222111643 (PMC8583884; doi:10.3390/ijms222111643)
Supplement: Supplementary file 1 [file ijms-22-11643-s001.zip › Kohlhase_IJMS_Supplemental_Figures.pdf]

Leaves

	g1	g2	g3	g4	g5	g6	g7	g8	g9	g10	g11	g12	g13	g14	g15	g16	g17	g18
g1	5147	305		121	1	5	1	2837		1	0	0	1	2		5	0	1
g2		639		135	3	4	0	215		0	0	0	0	2		0	0	1
g3																		
g4				559	1	3	0	70		0	0	0	2	0		0	0	3
g5					4	0	0	0		0	0	0	0	0		0	0	0
g6						8	0	5		0	0	0	0	0		0	0	0
g7							1	0		0	0	0	0	0		0	0	0
g8								6747		0	0	0	0	4		2	0	0
g9																		
g10										1	0	0	0	0		0	0	0
g11											5	0	0	0		2	0	0
g12												2	0	0		0	0	0
g13													4	0		0	0	0
g14														6		0	0	0
g15																		
g16																32	0	0
g17																	14	0
g18																		5

Roots

	g1	g2	g3	g4	g5	g6	g7	g8	g9	g10	g11	g12	g13	g14	g15	g16	g17	g18
g1	578																	
g2	57	566																
g3	112	134	619															
g4	74	103	106	251														
g5	28	111	58	66	217													
g6	10	18	15	17	21	77												
g7	22	53	31	27	45	27	186											
g8	30	44	56	30	35	26	49	415										
g9	4	5	5	5	7	10	10	7	16									
g10	14	28	23	21	24	25	43	43	6	232								
g11	12	21	18	16	14	25	25	23	9	11	113							
g12	48	34	72	31	22	22	43	48	6	22	21	483						
g13	35	26	33	18	23	15	32	32	7	27	20	41	1611					
g14	5	3	4	2	1	1	3	2	1	3	3	6	9	40				
g15	2	2	4	0	2	5	1	5	1	1	6	2	4	0	25			
g16	52	28	59	28	17	13	22	47	5	25	12	55	101	5	2	1526		
g17	16	16	21	15	8	12	17	11	7	8	12	20	31	2	4	19	237	
g18	26	25	32	21	13	16	27	27	8	18	23	26	29	3	9	43	28	199

Supplemental figure S1. Pairwise comparison of overlapping differentially expressed genes (DEGs) between 18 soybean genotypes. Significant DEGs (FDR < 0.05) responding after 60 minutes of iron stress were identified in leaf and root tissue of 18 soybean genotypes shown in black squares on the diagonals. Within each tissue type all combinations of genotype pairs were evaluated for DEG overlap. The blue color scale within each genotype matrix indicates the number of DEGs in common with dark blue squares indicating higher numbers of common DEGs between the two genotypes. Hierarchical cluster analysis based on phenotypic symptoms of iron stress revealed two major clusters of soybean genotypes, iron efficient and iron inefficient, indicated by green and grey squares, respectively. Genotypes 3, 9, and 15 in the leaf matrix (shown on the right) contain missing values due to sample removal during sequence processing.

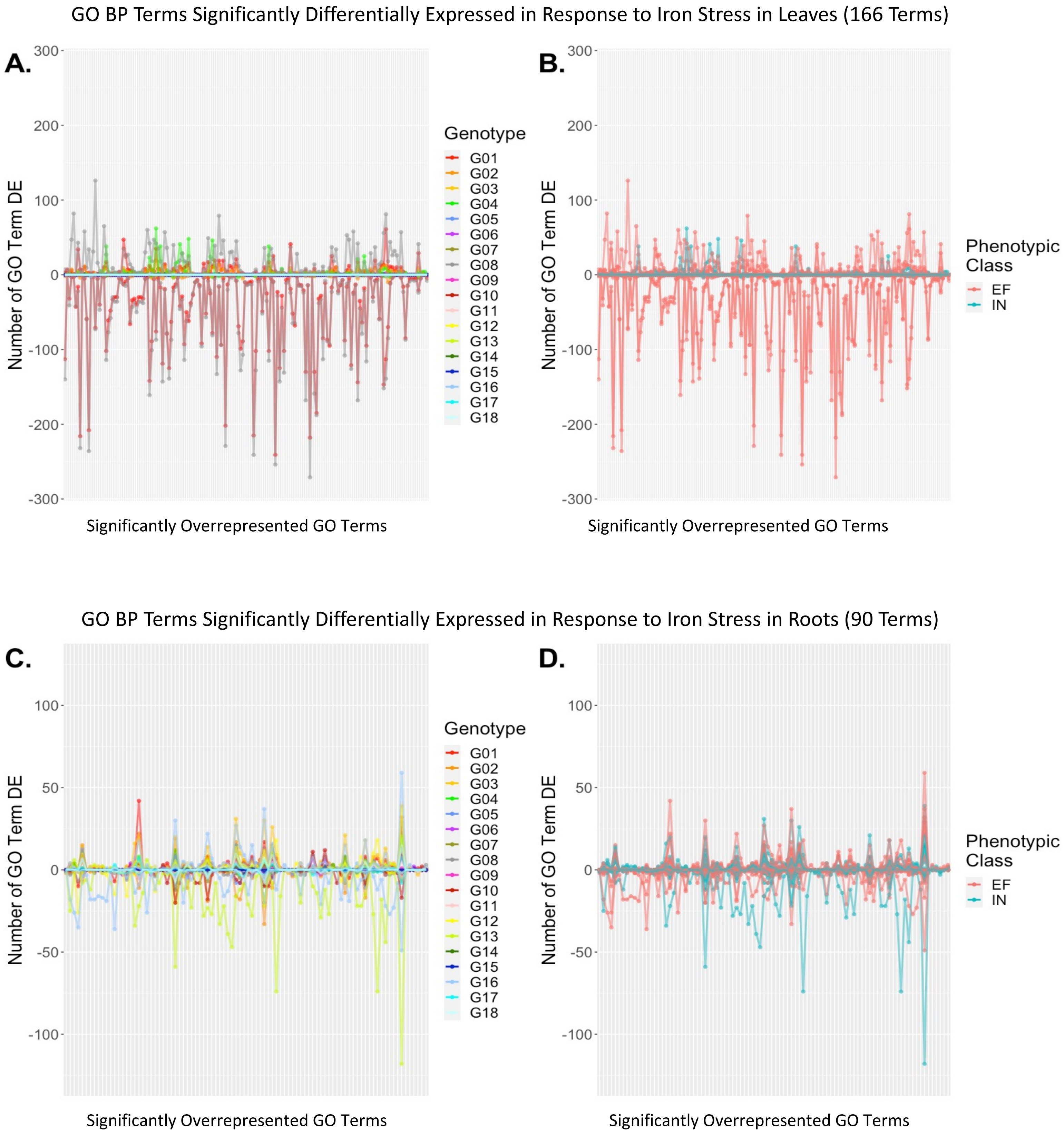

Supplemental figure S2. Number of differentially expressed genes (DEGs) associated with select gene ontology (GO) terms in leaf tissue of 18 soybean genotypes. GO term enrichment analysis was used on DEGs that were up-regulated or down-regulated in response to 60 minutes of iron stress in (A, B) leaf and (C, D) root tissue. GO terms that were significant in at least one genotype were compiled. The number of DEGs associated with each GO term were plotted for each genotype with up-regulated genes shown as positive values and down-regulated genes shown as negative values. (B, D) Previous hierarchical cluster analysis based on phenotypic measurements revealed two major clusters of soybean genotypes, iron efficient and iron inefficient, shown in red and blue, respectively.

Transcription factor families represented in response to iron stress in leaves (56 families)

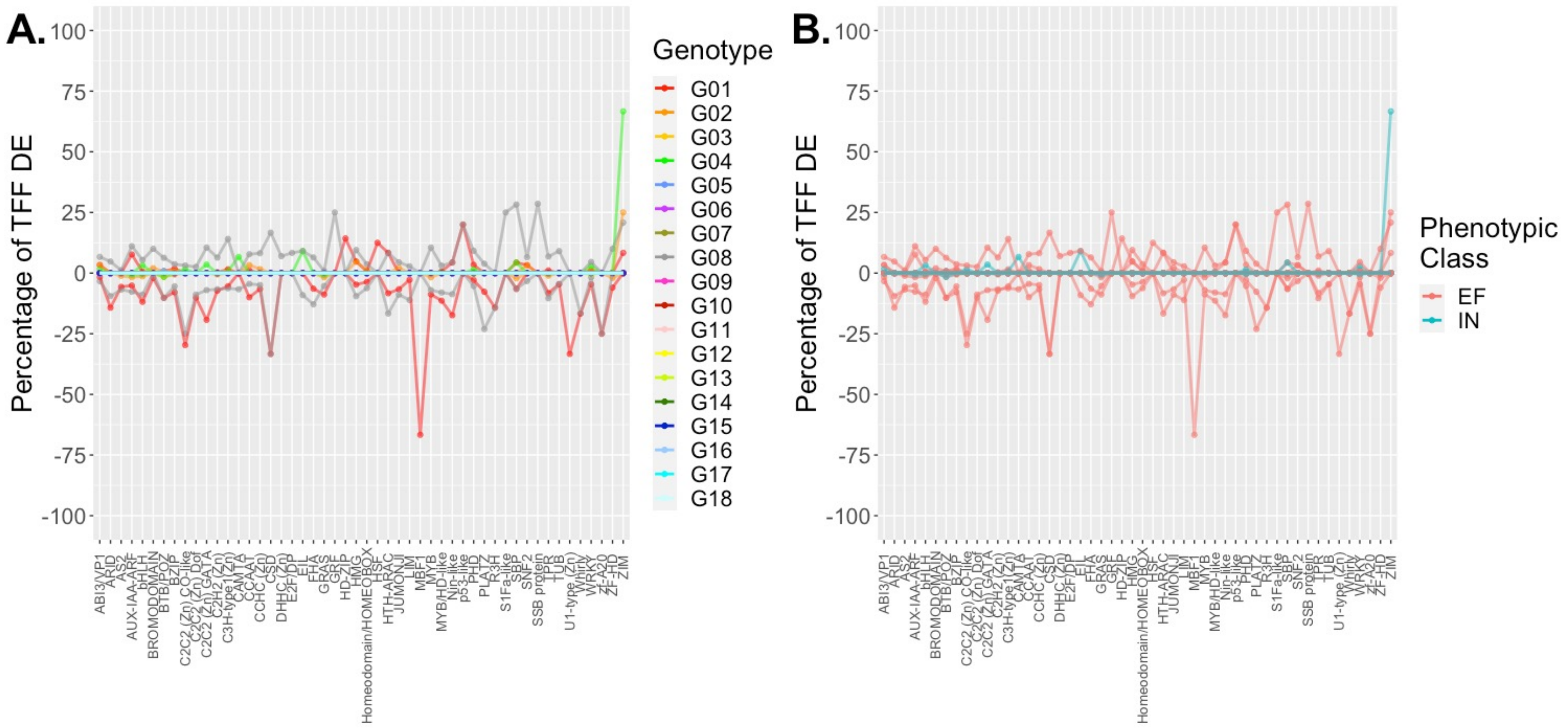

Transcription factor families represented in response to iron stress in roots (49 families)

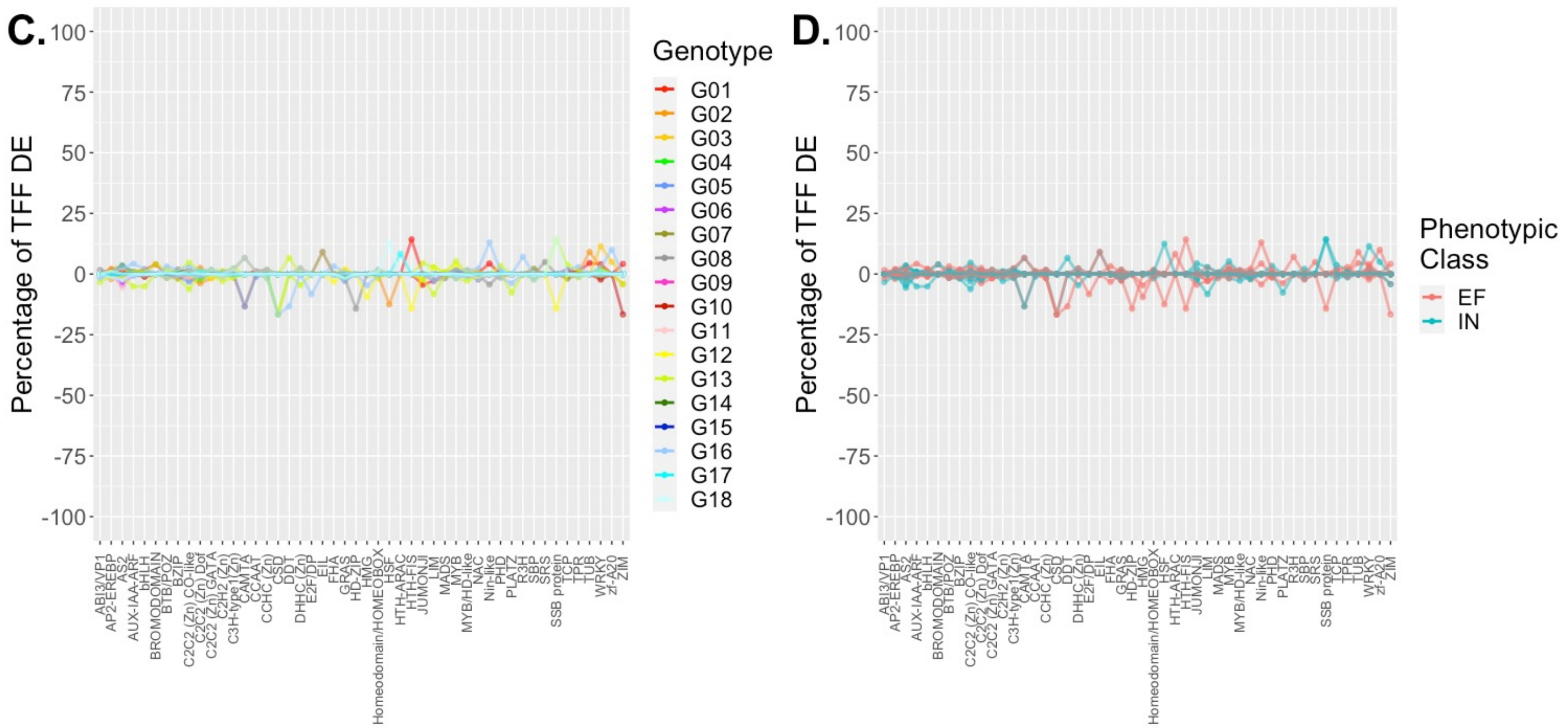

Supplemental figure S3. Differentially expressed transcription factor families in leaf tissue of 18 soybean genotypes. Differentially expressed genes (DEGs) were identified in (A, B) leaf and (C, D) root tissue in response to 60 minutes of iron stress. Transcription factors were identified in each DEG list and then numbers of genes were plotted by transcription factor family (TFF). The percentage relative to the total count of that TFF across the genome was calculated for each genotype with up-regulated genes shown as positive values and down-regulated genes shown as negative values. (B, D) Previous hierarchical cluster analysis based on phenotypic measurements revealed two major clusters of soybean genotypes, iron efficient and iron inefficient, shown in red and blue, respectively.

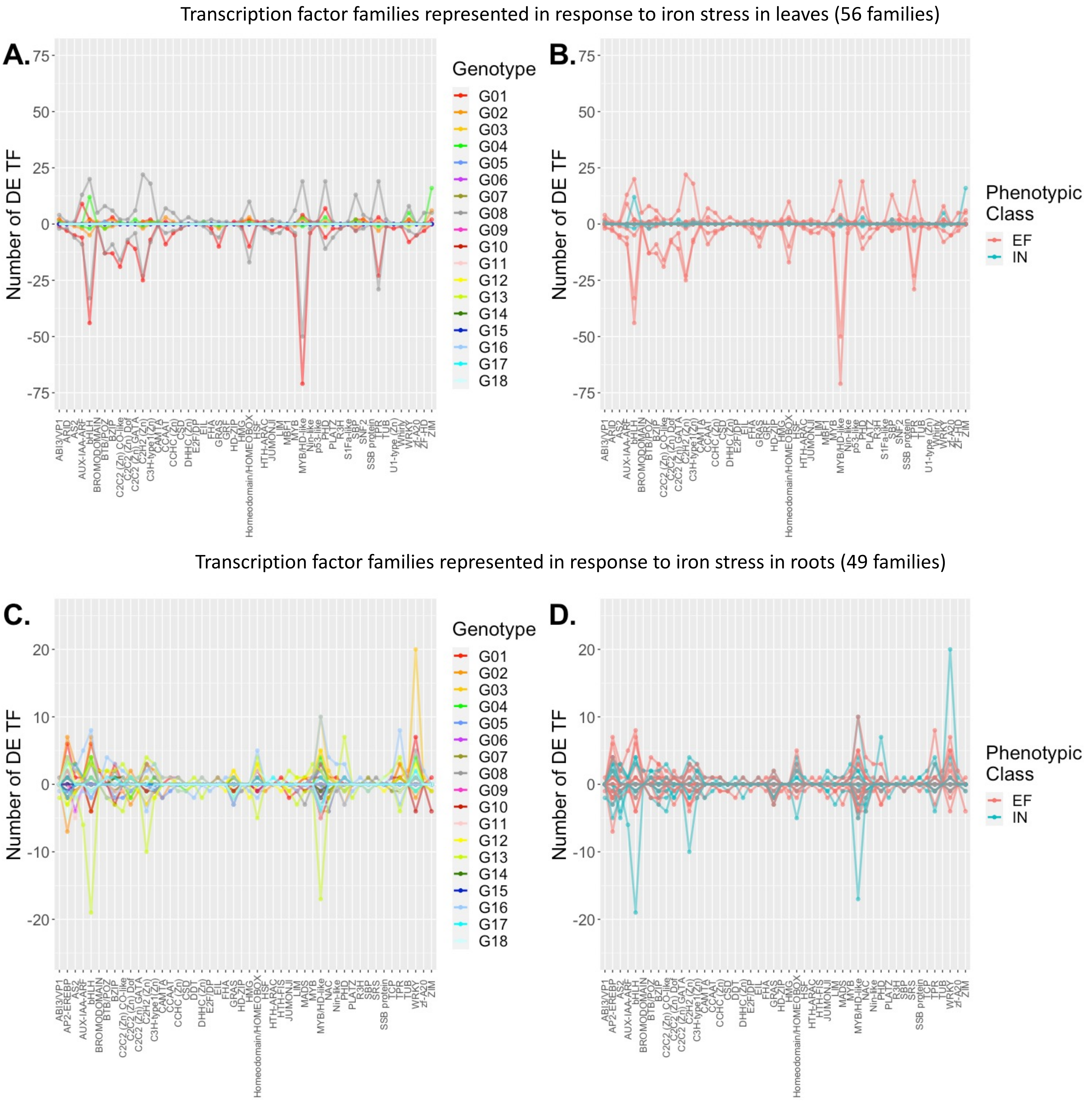

Supplemental figure S4. Differentially expressed transcription factor families in leaf tissue of 18 soybean genotypes. Differentially expressed genes (DEGs) were identified in (A, B) leaf and (C, D) root tissue in response to 60 minutes of iron stress. Transcription factors were identified in each DEG list and then numbers of genes were plotted by transcription factor family (TFF). The number of DEGs associated with each TFF were plotted for each genotype with up-regulated genes shown as positive values and down-regulated genes shown as negative values. (B, D) Previous hierarchical cluster analysis based on phenotypic measurements revealed two major clusters of soybean genotypes, iron efficient and iron inefficient, shown in red and blue, respectively.

A.

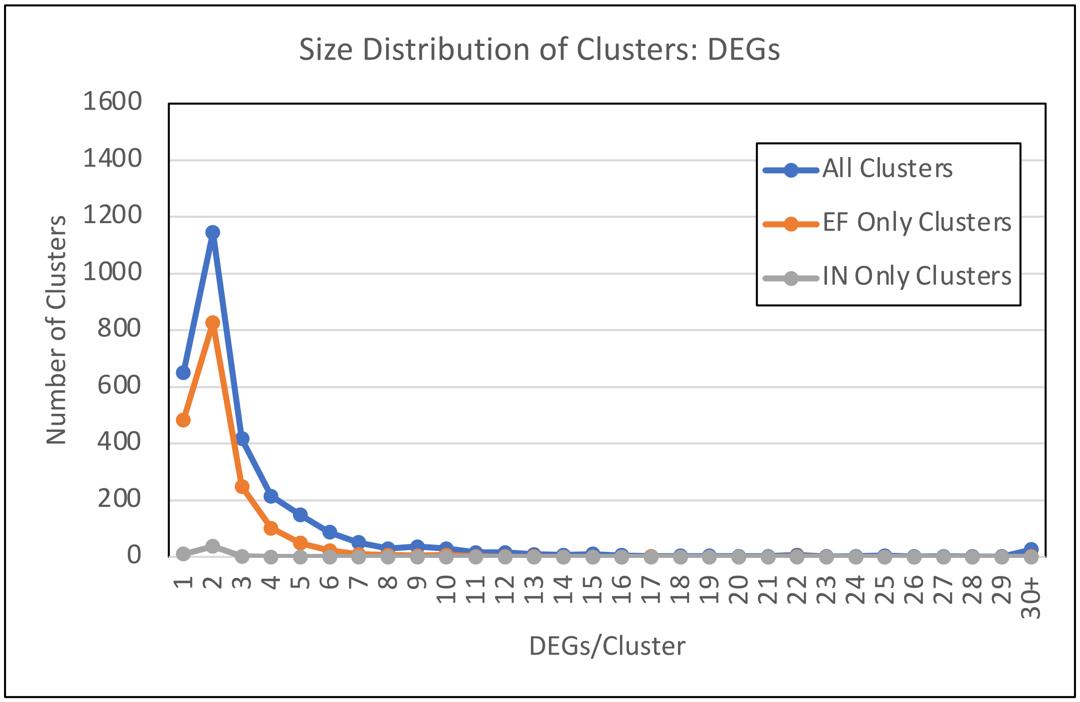

B.

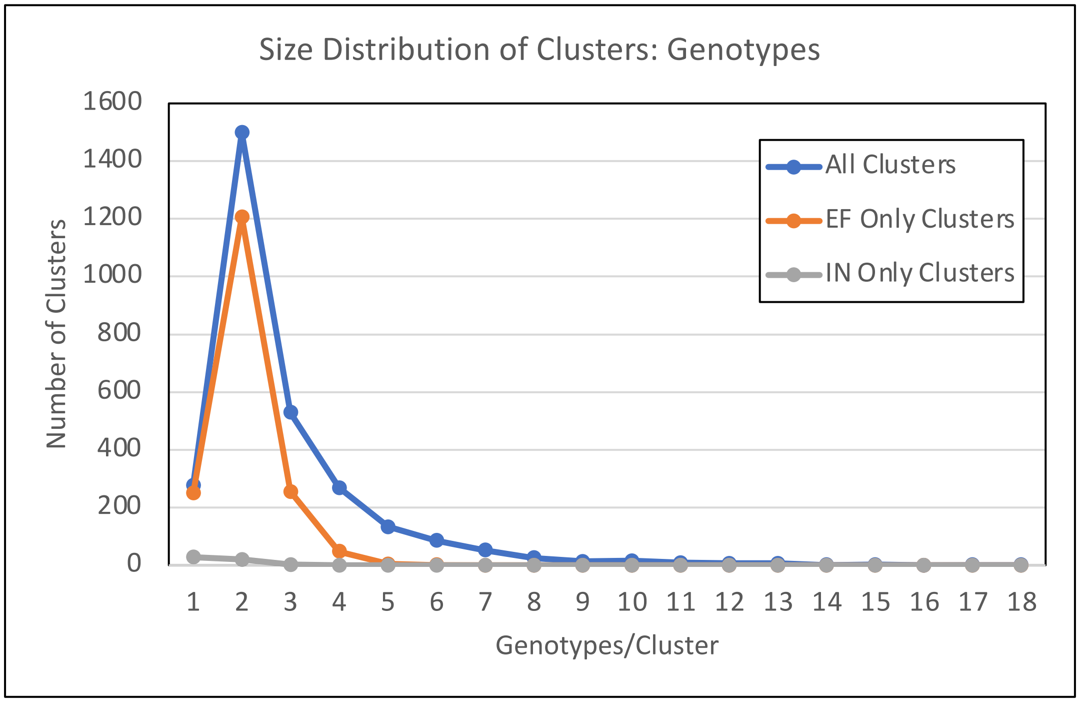

C.

	Average DEGs/Cluster	Average Genotypes/Cluster
All clusters	4.14 +/- 39.74	2.85 +/- 1.77
EF clusters	2.28 +/- 1.90	2.07 +/- 0.65
INF clusters	2.02 +/- 1.37	1.48 +/- 0.58

Supplemental figure S5. Distribution and summary of single linkage cluster analysis. Differentially expressed genes (DEGs) were identified across 18 soybean genotypes and two tissue types (leaves and roots) 60 minutes after iron stress. Single linkage clustering was used to identify DEGs with shared sequence homology. Previous hierarchical cluster analysis based on iron stress phenotypic measurements revealed two major clusters of soybean genotypes, iron efficient (EF) and iron inefficient (INF). The distributions of (A) DEGs per cluster and (B) genotypes per clusters were plotted for all clusters (blue), EF-specific clusters (orange), and INF-specific clusters (grey). (C) Average numbers of DEGs and genotypes per cluster were summarized (+/- standard deviation) for all clusters, EF-specific, and INF-specific clusters.
