# Supplementary material for: Comparing Early Transcriptomic Responses of 18 Soybean (Glycine max) Genotypes to Iron Stress
Source: Int J Mol Sci. 2021 Oct 28;22(21):11643. doi: 10.3390/ijms222111643 (PMC8583884; doi:10.3390/ijms222111643)
Supplement: Supplementary file 1 [file ijms-22-11643-s001.zip › Kohlhase_IJMS_Supplemental_Tables.pdf]

Plant Introduction ID	Genotype	DEGs in Leaves			DEGs in Roots			Phenotypic Response Cluster
		Down	Total	Up	Down	Total	Up	
PI 437377	G1	4224	5147	923	117	578	461	EF
PI 612611	G2	212	639	427	209	566	357	EF
PI 578366	G3	-	-	-	98	619	521	INF
PI 464877	G4	102	559	457	83	251	168	INF
PI 438103	G5	1	4	3	125	217	92	INF
PI 437462A	G6	0	8	8	42	77	35	INF
PI 196150	G7	1	1	0	107	186	79	INF
PI 153280	G8	4268	6747	2479	144	415	271	EF
PI 092465	G9	-	-	-	12	16	4	INF
PI 090392	G10	0	1	1	133	232	99	EF
PI 089156	G11	4	5	1	89	113	24	INF
PI 086145	G12	2	2	0	204	483	279	EF
PI 084973	G13	4	4	0	1222	1611	389	INF
PI 080459	G14	2	6	4	13	40	27	EF
PI 068685	G15	-	-	-	12	25	13	EF
PI 054854	G16	25	32	7	759	1526	767	EF
PI 548533	G17 (Clk)	1	14	13	86	237	151	EF
PI 547430	G18 (Iso)	4	5	1	70	199	129	INF

Supplemental table S1: Numbers of differentially expressed genes (DEGs) for 18 soybean genotypes. Significant DEGs (FDR < 0.05) were identified in the leaves and roots 60 minutes after iron stress and are presented as down-regulated, total, or up-regulated DEGs. Missing values for genotypes G3, G9, and G15 in leaves were due to sample removal during sequence processing. Phenotypic Response Cluster refers to the two major groups of soybeans, iron efficient (EF) or iron inefficient (INF), identified by clustering phenotypic measurements of iron stress response.

Number of genotypes sharing the same DEG	Number of DEGs					
	Leaves			Roots		
	All	EF	INF	All	EF	INF
17	-	-	-	1	-	-
16	-	-	-	0	-	-
15	0	-	-	1	-	-
14	0	-	-	4	-	-
13	0	-	-	0	-	-
12	0	-	-	1	-	-
11	0	-	-	2	-	-
10	0	-	-	3	-	-
9	0	-	-	4	0	0
8	0	0	-	8	0	0
7	0	0	0	5	0	0
6	0	0	0	17	0	0
5	2	0	0	32	0	1
4	24	1	0	78	0	1
3	192	123	0	193	12	9
2	2,992	2,859	4	668	159	77
1	6,508	6,158	350	4,615	2,677	1,938
Total (unique)	9,718	9,141	354	5,632	2,848	2,026

Supplemental table S2: Number of differentially expressed genes (DEGs) identified in multiple soybean genotypes. Significant DEGs (FDR < 0.05) responding after 60 minutes of iron stress were identified within each genotype x tissue type combination then cross referenced across 15 soybean genotypes in leaf tissue or 18 soybean genotypes in root tissue. The number of genes differentially expressed in only iron efficient or inefficient genotypes are identified in the EF and INF columns, respectively.

Genotype	Leaves	Roots
G1	75	2
G2	0	9
G3	-	4
G4	15	5
G5	0	4
G6	0	4
G7	0	1
G8	86	1
G9	-	0
G10	0	8
G11	1	2
G12	0	5
G13	0	26
G14	0	0
G15	-	0
G16	1	3
G17	0	0
G18	0	0

Supplemental table S3: Numbers of overrepresented gene ontology (GO) terms identified in 18 soybean genotypes. Significantly overrepresented GO terms (corrected p-value < 0.05) were identified using GO terms associated with differentially expressed genes responding after 60 minutes of iron stress in the leaves and roots for each genotype.

Number of genotypes sharing the same overrepresented GO term	Number of overrepresented GO terms	
	Leaves	Roots
6	0	1
5	0	0
4	0	2
3	0	0
2	72	9
1	34	42
Total (unique)	106	54

Supplemental table S4: Number of overrepresented gene ontology (GO) terms identified in multiple soybean genotypes. Significantly overrepresented GO terms (corrected p-value < 0.05) were identified from differentially expressed gene lists for leaf and root tissue of 18 soybean genotypes after 60 minutes of iron stress. Overrepresented GO terms were cross references across genotypes within each tissue type.

Genotype	Leaves		Roots	
	TFs	Overrepresented TFFs	TFs	Overrepresented TFFs
G1	433	2	54	0
G2	59	1	85	0
G3	-	-	78	1
G4	66	1	36	0
G5	0	0	36	0
G6	1	0	12	1
G7	0	0	18	0
G8	620	2	41	0
G9	-	-	3	0
G10	0	0	35	1
G11	0	0	15	1
G12	0	0	47	0
G13	2	0	150	0
G14	0	0	5	0
G15	-	-	3	0
G16	1	0	126	0
G17	1	0	21	0
G18	0	0	32	0

Supplemental table S5: Number of differentially expressed transcription factors (TFs) and significantly overrepresented transcription factor families (TFFs). Differentially expressed genes were identified in response to 60 minutes of iron stress in leaves and roots of 18 soybean genotypes. TF enrichment analysis was used to identify significantly overrepresented TFFs (corrected p-value < 0.05) within each genotype x tissue TF list.

**Number of overlapping experimental conditions (experiment x timepoint x tissue type)**	**Number of DEGs**	
	**Leaves**	**Roots**
8	3	0
7	15	5
6	41	53
5	118	99
4	341	196
3	900	598
2	1,614	1,055
1	2,459	1,478
Total (unique)	5,491	3,493

Supplemental table S6: Number of differentially expressed genes (DEGs) identified in multiple iron stress experimental conditions. Significant DEGs (FDR < 0.05) responding after 60 minutes of iron stress were identified within each genotype x tissue type combination then cross referenced with Clark gene expression studies conducted by Moran Lauter et al. (2014), Moran Lauter et al. (2020), Atencio et al. (2021), and O'Rourke et al. (2019). These experiments ranged from 30 minutes up to 10 days of iron stress and examine differential expression in leaves and roots.

Number of genotypes sharing the same DEG (current study)

Experimental conditions with same DEG (past studies)

	1	2	3	4	5	6	7	8	Total
1	1,638	1,088	532	186	56	18	9	1	3,528
2	775	491	346	142	58	20	6	2	1,840
3	38	27	20	13	4	3	0	0	105
4	8	8	2	0	0	0	0	0	18
Total	2,459	1,614	900	341	118	41	15	3	5,491

Supplemental table S7: Contingency table of differentially expressed genes (DEGs) identified in leaves of multiple soybean genotypes and multiple iron stress experimental conditions. In leaves, significant DEGs (FDR < 0.05) responding after 60 minutes of iron stress were identified within each genotype, compiled, then cross referenced with Clark gene expression studies conducted by Moran Lauter et al. (2014), Moran Lauter et al. (2020), Atencio et al. (2021), and O'Rourke et al. (2019). These experiments ranged from 30 minutes up to 10 days of iron stress and examine differential expression in leaves and roots.

Experimental conditions with same DEG (past studies)

Number of genotypes sharing the same DEG (current study)

	1	2	3	4	5	6	7	Total
1	1,188	813	475	153	84	48	4	2,765
2	183	166	78	27	12	4	0	470
3	60	36	26	12	3	0	1	138
4	24	20	11	2	0	0	0	57
5	13	6	3	0	0	0	0	22
6	7	5	3	0	0	0	0	15
7	2	2	0	0	0	1	0	5
8	3	1	1	1	0	0	0	6
9	3	0	1	0	0	0	0	4
10	1	1	0	1	0	0	0	3
11	1	0	0	0	0	0	0	1
12	1	0	0	0	0	0	0	1
13	0	0	0	0	0	0	0	0
14	0	4	0	0	0	0	0	4
15	0	1	0	0	0	0	0	1
16	0	0	0	0	0	0	0	0
17	1	0	0	0	0	0	0	1
Total	1,487	1,055	598	196	99	53	5	3,493

Supplemental table S8: Contingency table of differentially expressed genes (DEGs) identified in roots of multiple soybean genotypes and multiple iron stress experimental conditions. In roots, significant DEGs (FDR < 0.05) responding after 60 minutes of iron stress were identified within each genotype, compiled, then cross referenced with Clark gene expression studies conducted by Moran Lauter et al. (2014), Moran Lauter et al. (2020), Atencio et al. (2021), and O'Rourke et al. (2019). These experiments ranged from 30 minutes up to 10 days of iron stress and examine differential expression in leaves and roots.
